# Hawaii’s “7 by 7” for School Health Education: A PowerPoint Presentation on Integrating the National Health Education Standards With Priority Content Areas for Today’s School Health Education in Grades Kindergarten Through 12

**Published:** 2006-03-15

**Authors:** Beth Pateman

## Abstract

School-based health education can help young people develop the knowledge, skills, motivation, and support they need to choose health-enhancing behaviors and resist engaging in behaviors that put them at risk for health and social problems and school failure. The health of school-age youth is significantly associated with their school achievement. However, in the midst of today's increased emphasis on school accountability in the areas of reading, writing, and mathematics, subject areas such as health education tend to receive less prominence in the school curriculum. Recalling their own lackluster school experiences related to health topics, decision makers may not realize that today's skills-based school health curriculum involves a highly interactive and engaging approach to promoting good health and preventing the most serious health problems among youth. Health education is one important component of a coordinated school health program that includes health education, physical education, school health services, nutrition services, school counseling and psychological services, a healthy school environment, school promotion for faculty and staff, and involvement of family and community members. The purpose of this PowerPoint presentation — Healthy Keiki, Healthy Hawaii: Hawaii's "7 by 7" for School Health Education — is to educate health and education decision makers, teachers, parents, and community members on how Hawaii has integrated seven health education standards with seven priority health content areas to create an effective approach to school health education in grades kindergarten through 12. The goal of Hawaii's "7 by 7" curriculum focus is to ensure that all of Hawaii's *keiki* (children) have well-planned opportunities at school to become fit, healthy, and ready to learn.

## Download Healthy Keiki, Healthy Hawaii: Hawaii's "7 by 7" for School Health Education

**Figure:**
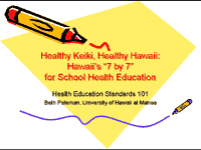

## Information Sources

- **Healthy Youth:** http://www.cdc.gov/HealthyYouth/ Division of Adolescent and School Health, National Center for Chronic Disease Prevention and Health Promotion, Centers for Disease Control and Prevention, U.S. Department of Health and Human Services
- **Kidshealth:** http://www.kidshealth.org The Nemours Foundation's Center for Children's Health Media
- **Health Finder for Kids:** http://www.healthfinder.gov/kids Office of Disease Prevention and Health Promotion, U.S. Department of Health and Human Services
- **BAM! Body and Mind:** http://www.bam.gov/ Centers for Disease Control and Prevention, U.S. Department of Health and Human Services
- **American School Health Association:** http://www.ashaweb.org
- **American Association for Health Education:** http://www.aahperd.org/aahe
- **American Cancer Society:** http://www.cancer.org
- **Rocky Mountain Center for Health Promotion and Education:** http://www.rmc.org
- **HealthTeacher:** http://www.healthteacher.com

