# Supplementary material for: Hawaii’s “7 by 7” for School Health Education: A PowerPoint Presentation on Integrating the National Health Education Standards With Priority Content Areas for Today’s School Health Education in Grades Kindergarten Through 12
Source: Prev Chronic Dis. 2006 Mar 15;3(2):A63. (PMC1563955)
Supplement: Supplementary file 1 [file 05_0098_01.ppt]

## Slide 1
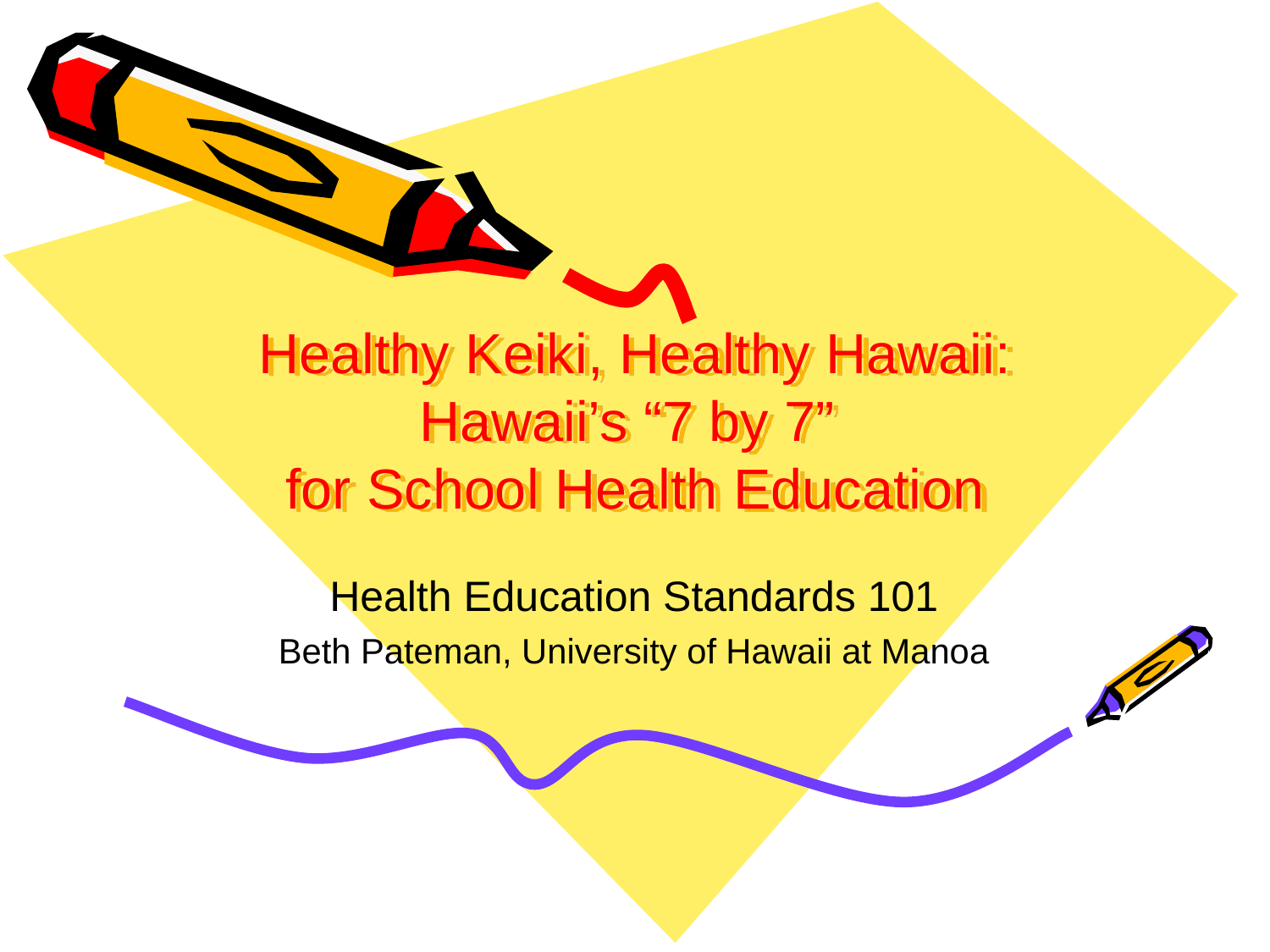

# Healthy Keiki, Healthy Hawaii: Hawaii’s “7 by 7” for School Health Education
Health Education Standards 101
Beth Pateman, University of Hawaii at Manoa

## Slide 2
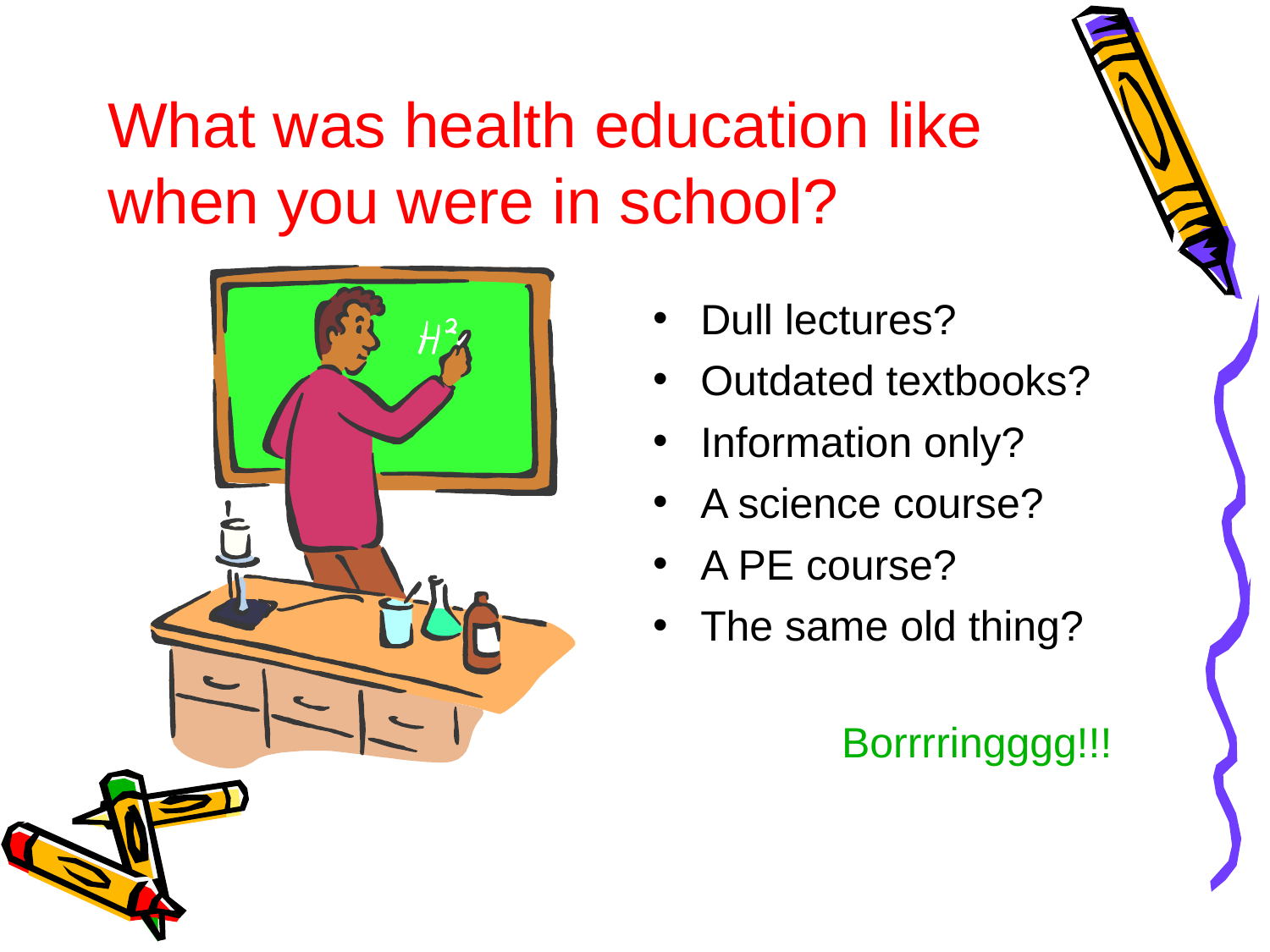

# What was health education like when you were in school?
Dull lectures?
Outdated textbooks?
Information only?
A science course?
A PE course?
The same old thing?
 Borrrringggg!!!

## Slide 3
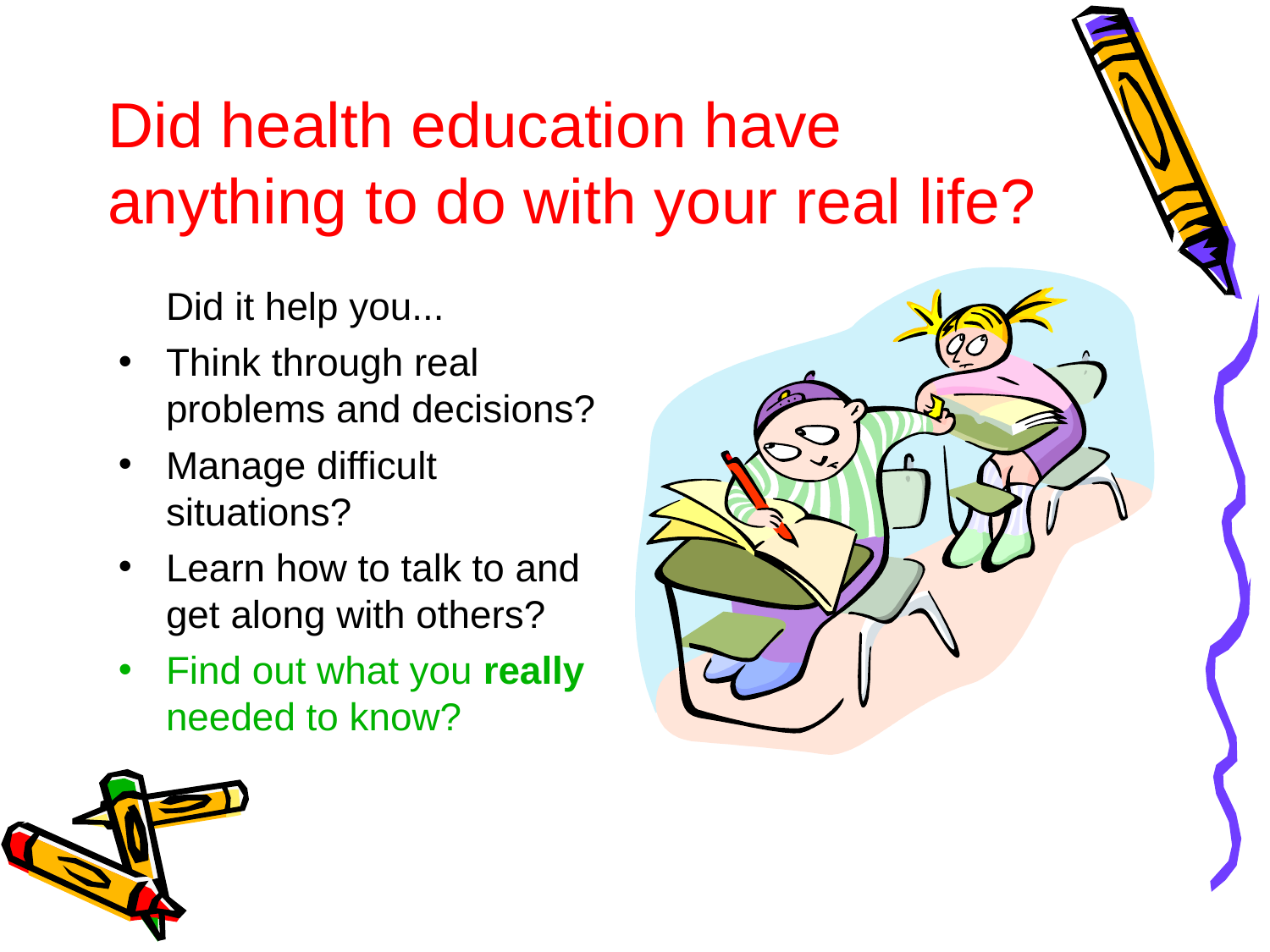

# Did health education have anything to do with your real life?
Did it help you...
Think through real problems and decisions?
Manage difficult situations?
Learn how to talk to and get along with others?
Find out what you really needed to know?

## Slide 4
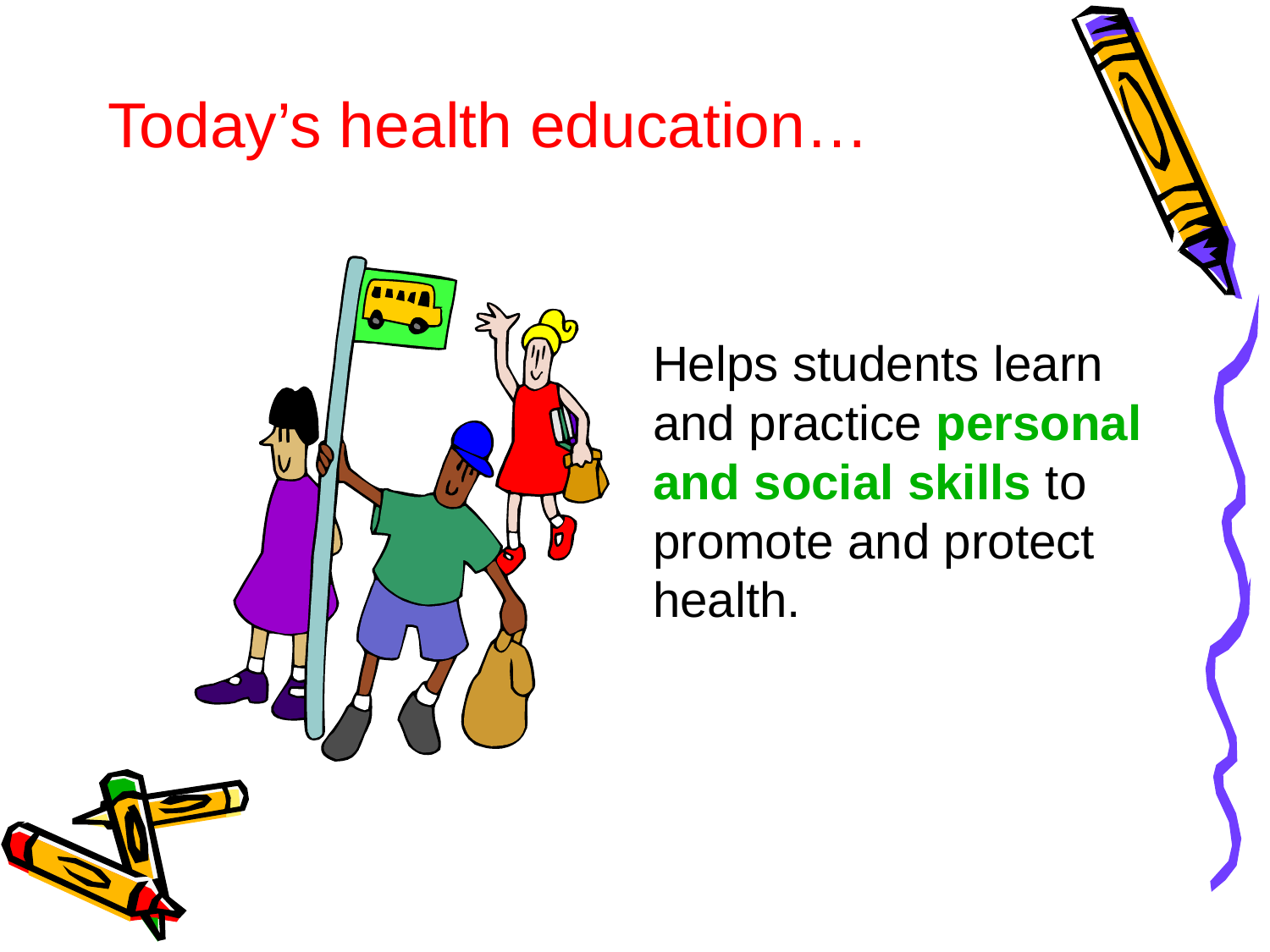

Today’s health education…
# Helps students learn and practice personal and social skills to promote and protect health.

## Slide 5
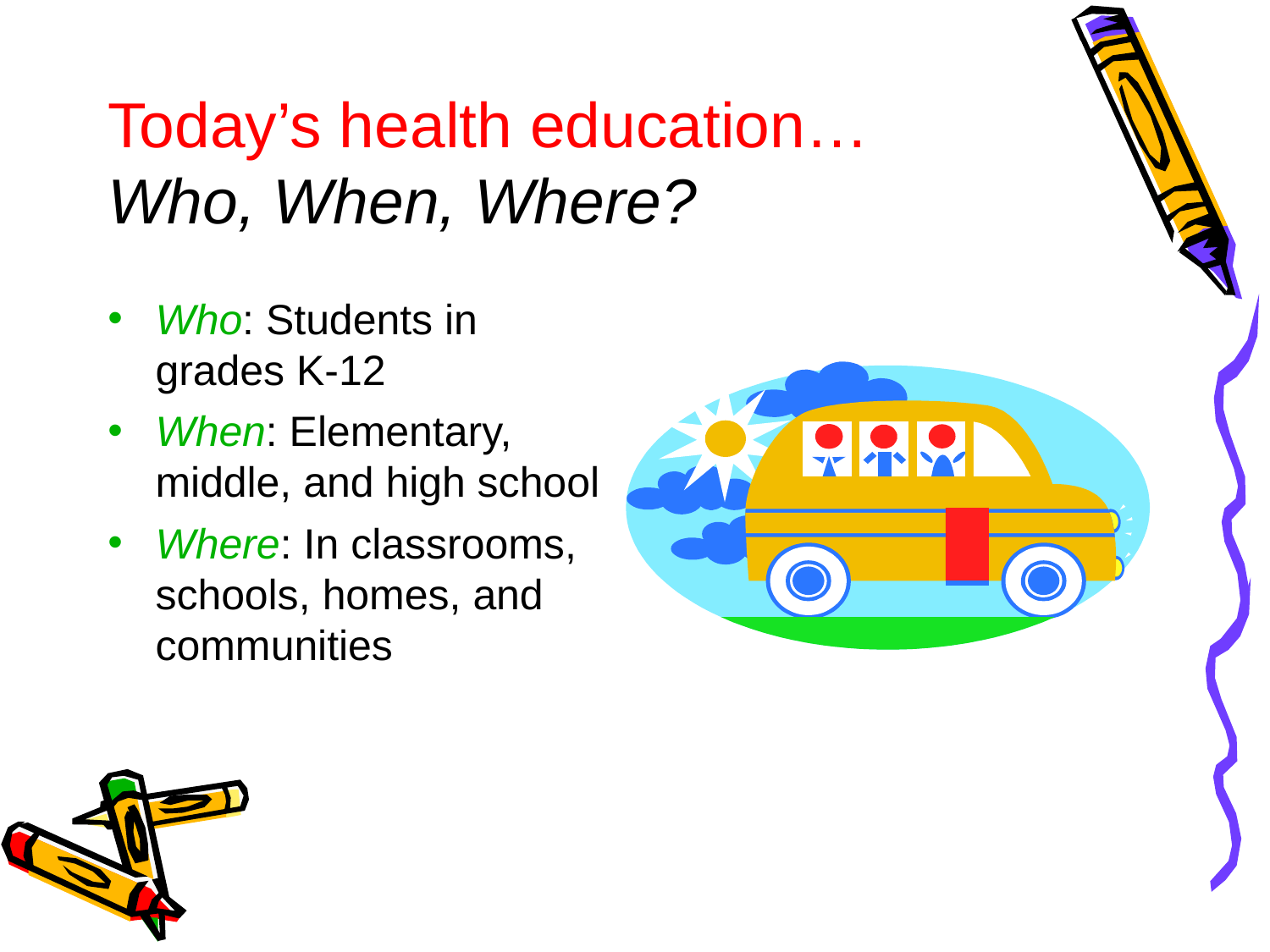

Today’s health education…
# Who, When, Where?
Who: Students in grades K-12
When: Elementary, middle, and high school
Where: In classrooms, schools, homes, and communities
Healthy Keiki, Health Hawaii

## Slide 6
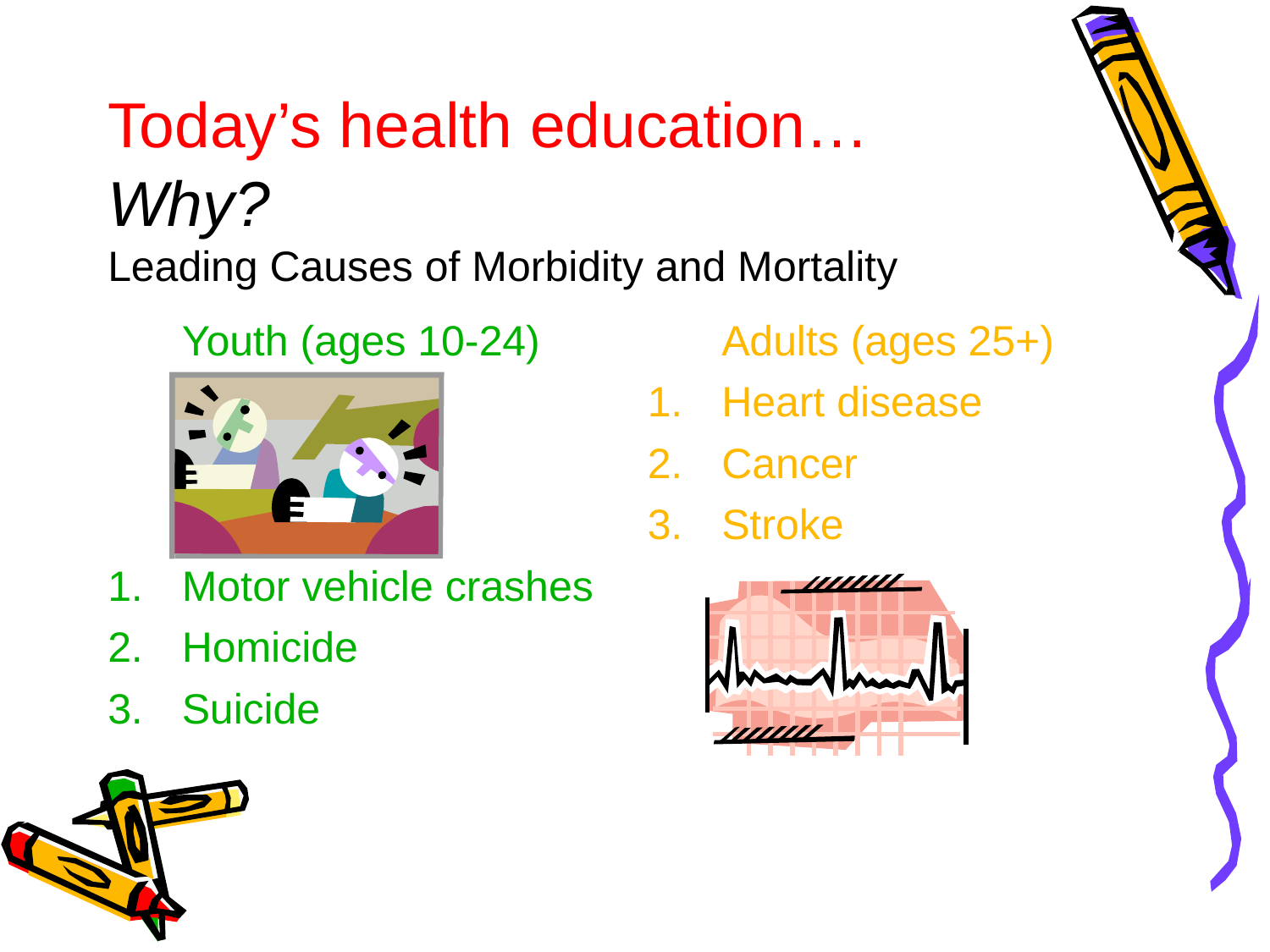

Today’s health education…
# Why? Leading Causes of Morbidity and Mortality
Youth (ages 10-24)
Motor vehicle crashes
Homicide
Suicide
Adults (ages 25+)
Heart disease
Cancer
Stroke

## Slide 7
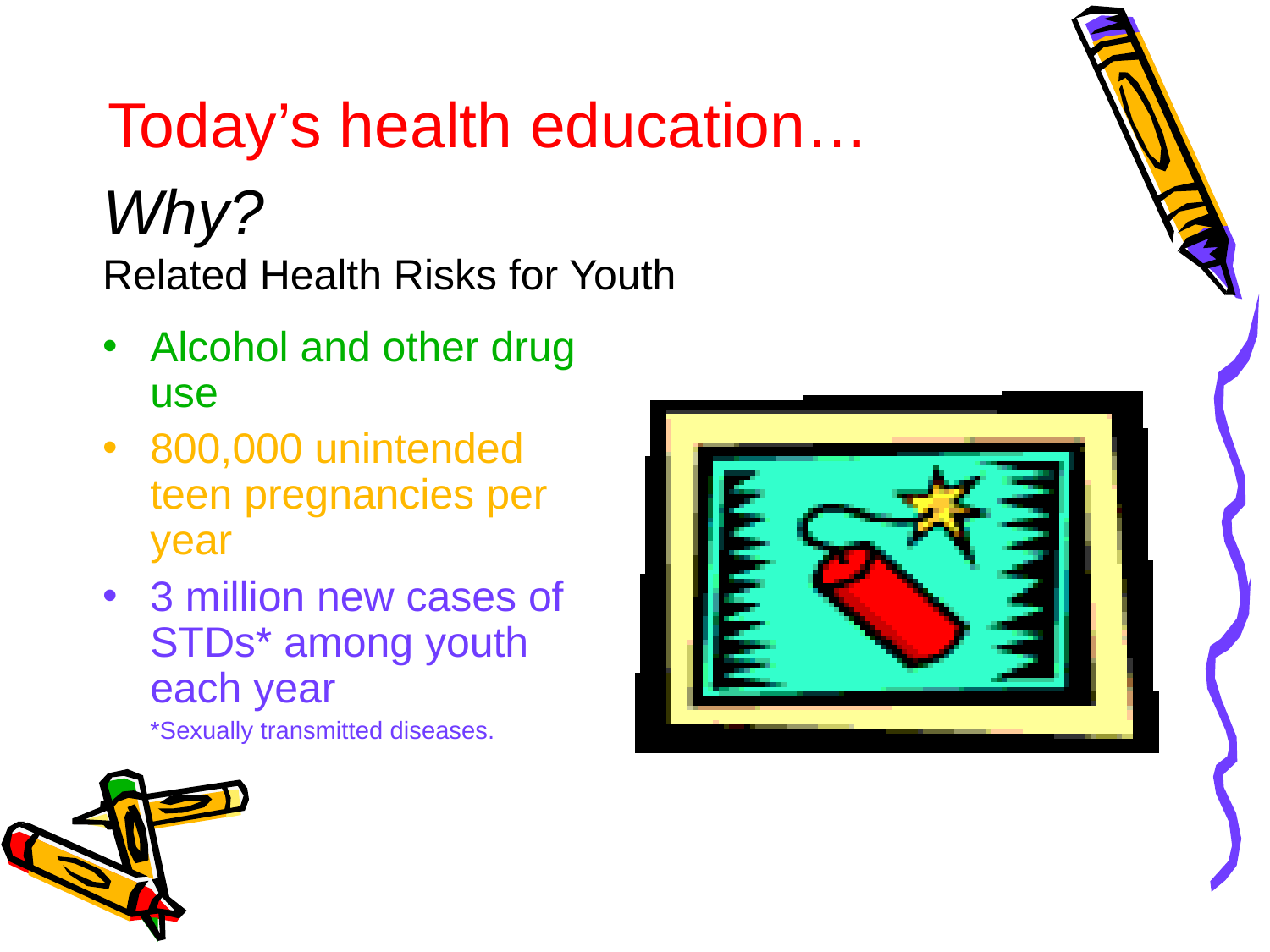

Today’s health education…
# Why? Related Health Risks for Youth
Alcohol and other drug use
800,000 unintended teen pregnancies per year
3 million new cases of STDs* among youth each year
*Sexually transmitted diseases.

## Slide 8
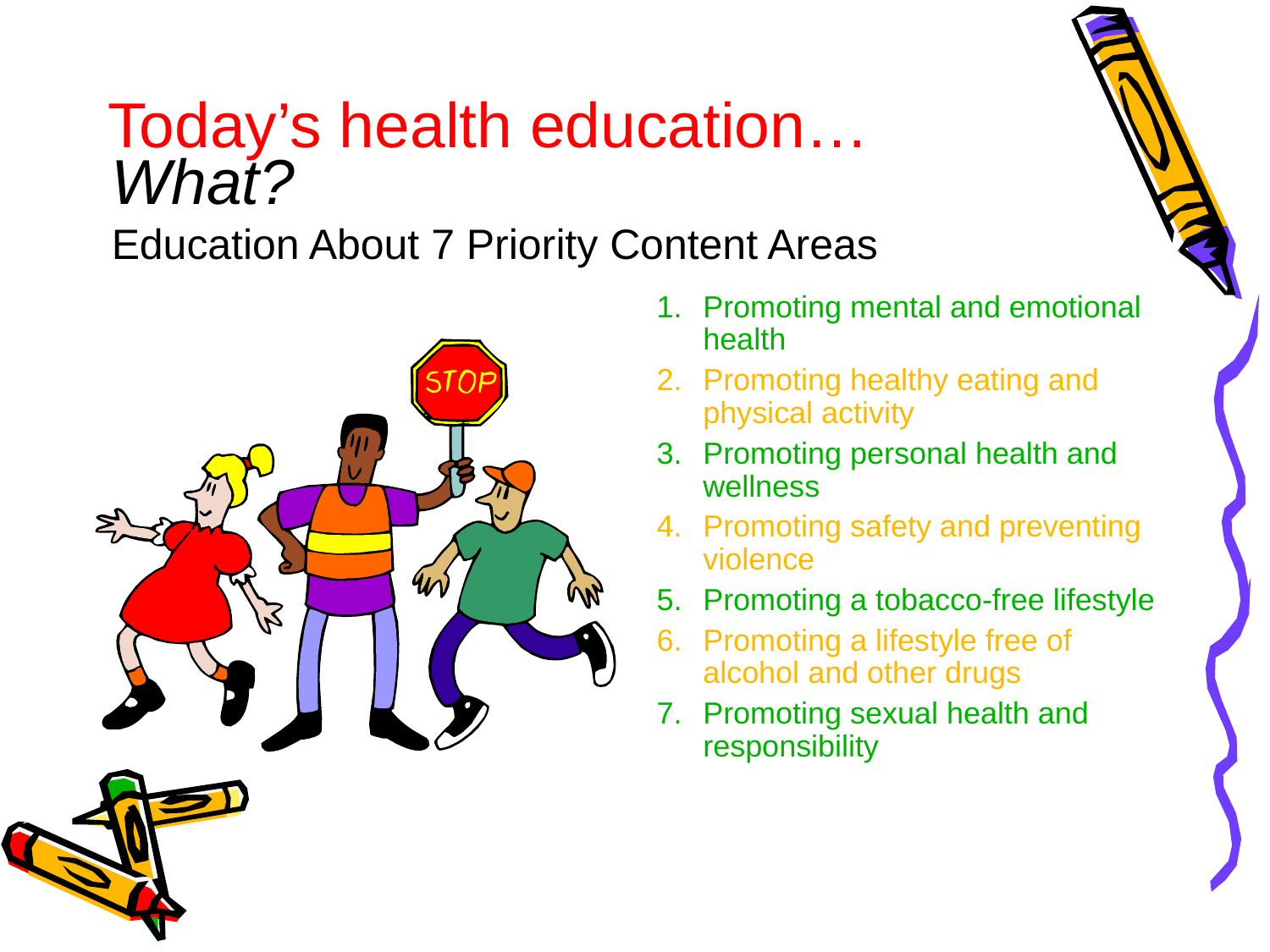

# What? Education About 7 Priority Content Areas
Today’s health education…
Promoting mental and emotional health
Promoting healthy eating and physical activity
Promoting personal health and wellness
Promoting safety and preventing violence
Promoting a tobacco-free lifestyle
Promoting a lifestyle free of alcohol and other drugs
Promoting sexual health and responsibility

## Slide 9
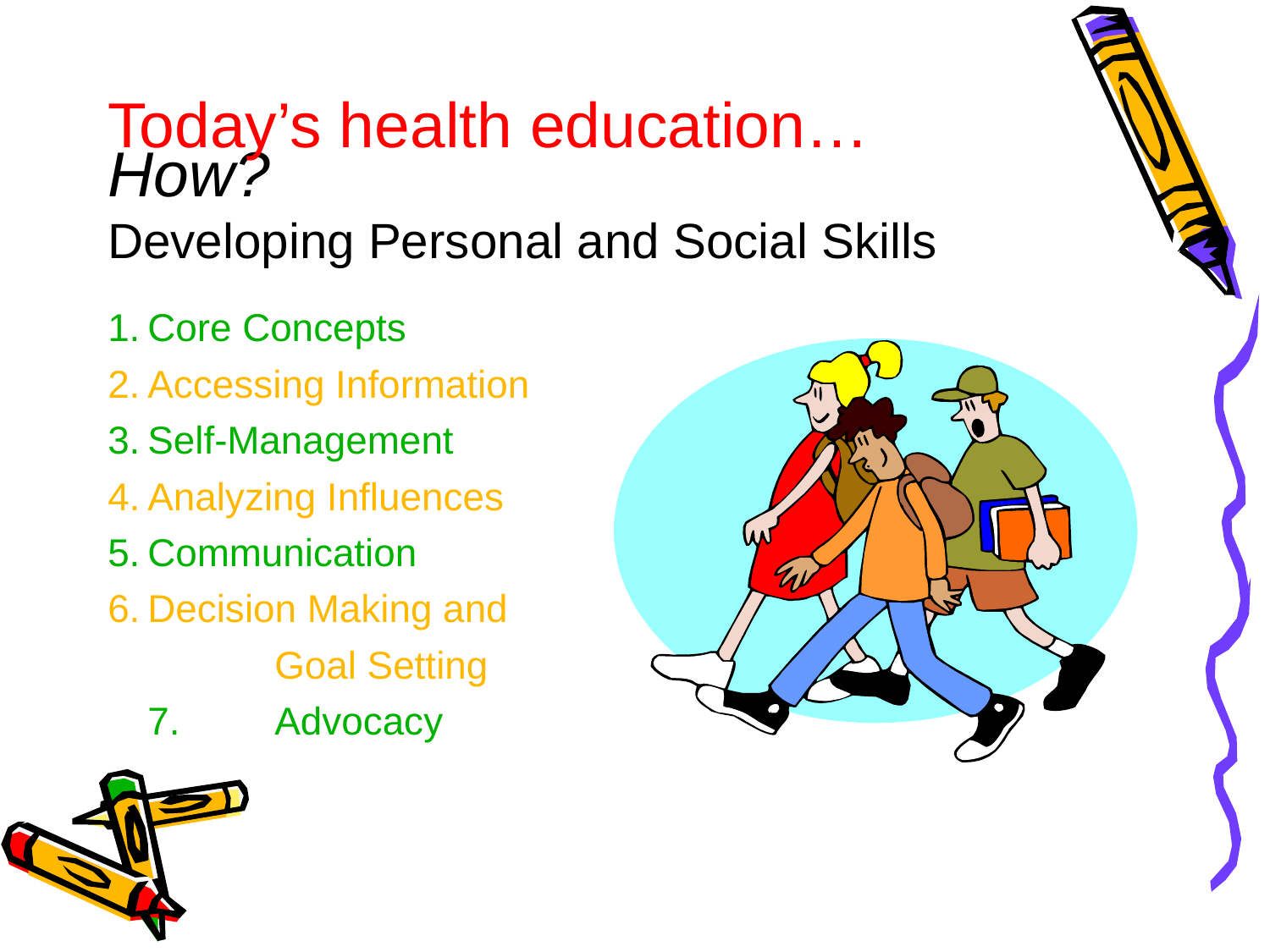

# How? Developing Personal and Social Skills
Today’s health education…
Core Concepts
Accessing Information
Self-Management
Analyzing Influences
Communication
Decision Making and
	Goal Setting
7.	Advocacy

## Slide 10
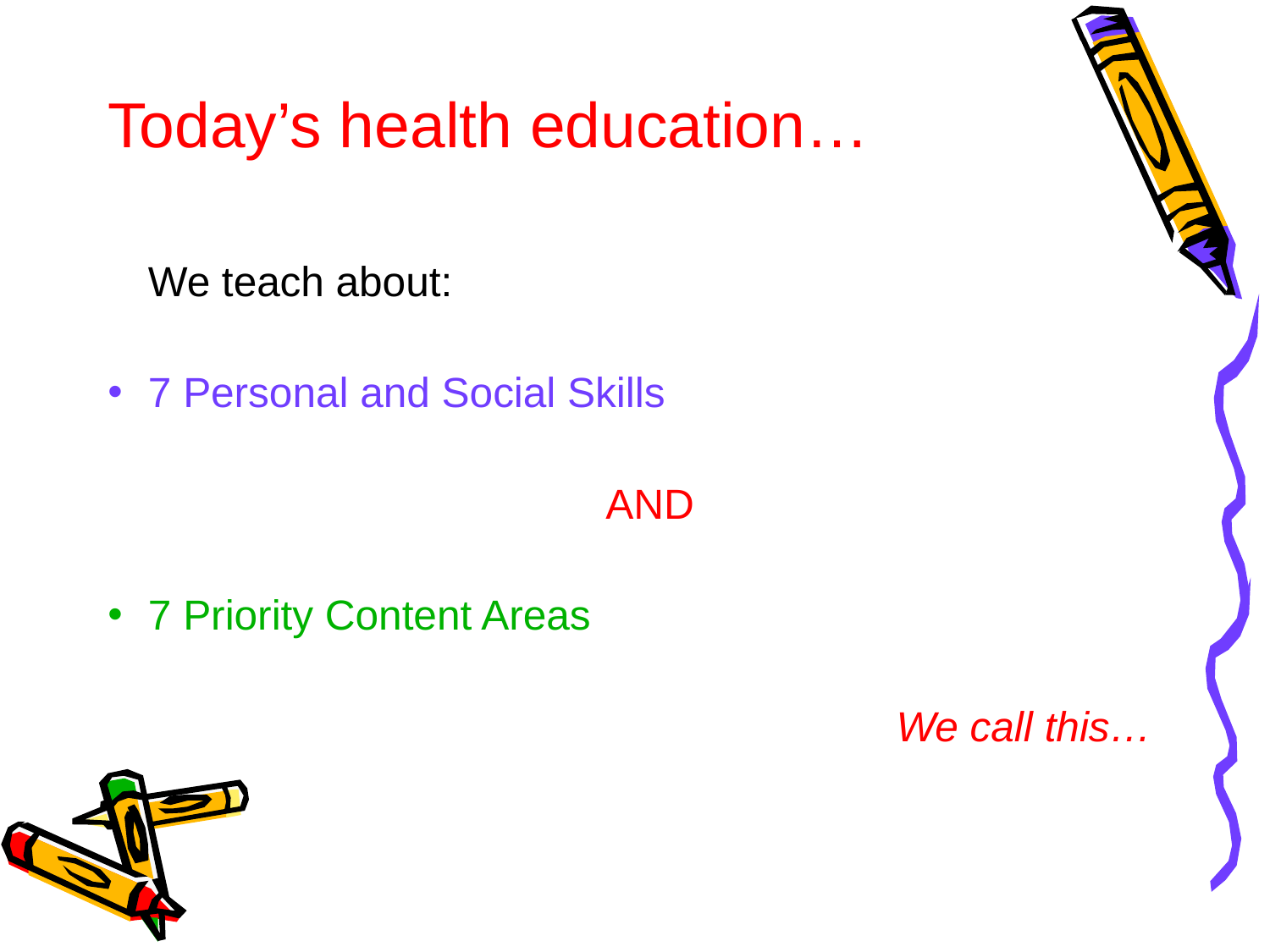

Today’s health education…
# We teach about:
7 Personal and Social Skills
AND
7 Priority Content Areas
We call this…

## Slide 11
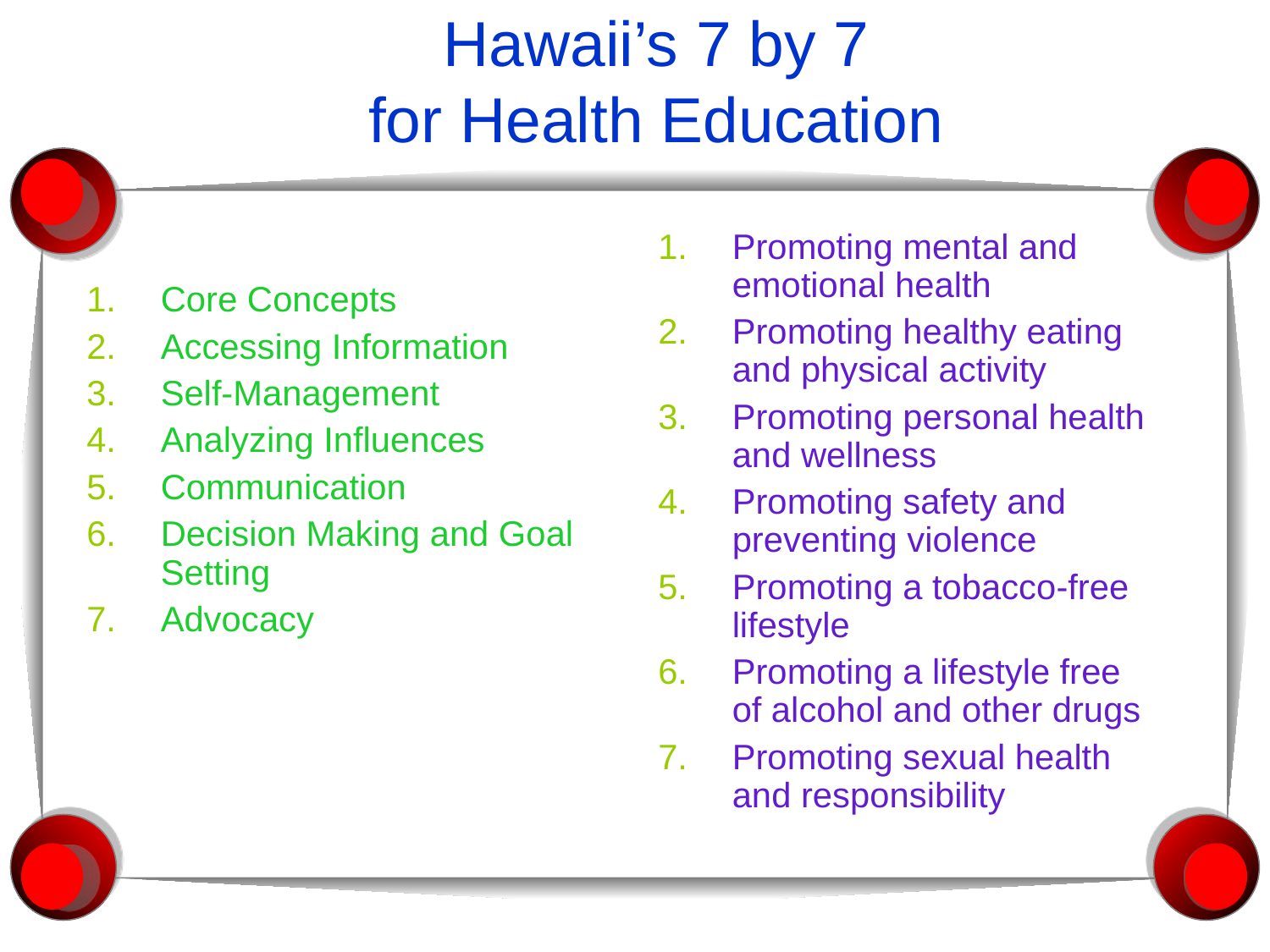

# Hawaii’s 7 by 7 for Health Education
Promoting mental and emotional health
Promoting healthy eating and physical activity
Promoting personal health and wellness
Promoting safety and preventing violence
Promoting a tobacco-free lifestyle
Promoting a lifestyle free of alcohol and other drugs
Promoting sexual health and responsibility
Core Concepts
Accessing Information
Self-Management
Analyzing Influences
Communication
Decision Making and Goal Setting
Advocacy

## Slide 12
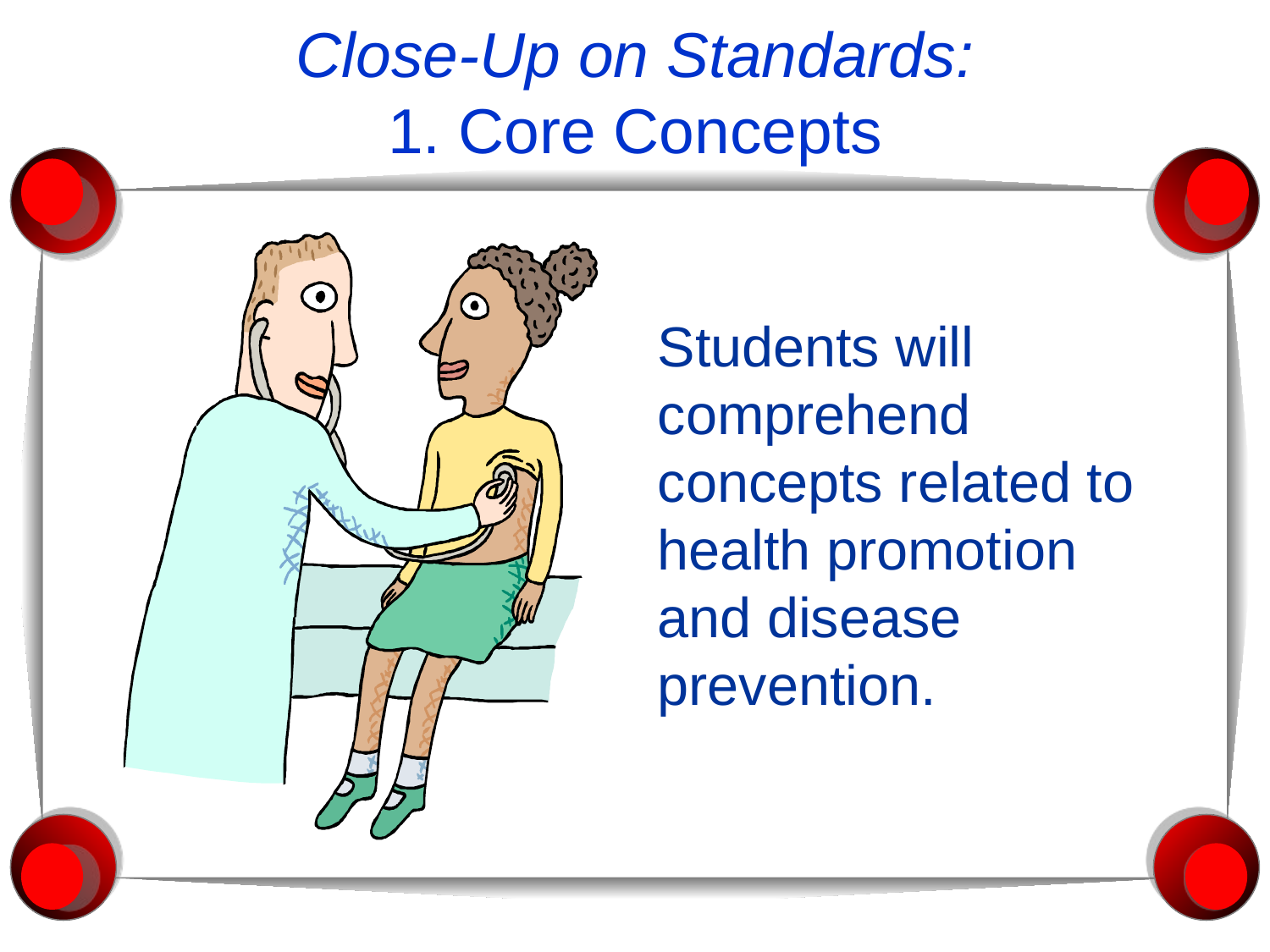

# Close-Up on Standards: 1. Core Concepts
Students will comprehend concepts related to health promotion and disease prevention.

## Slide 13
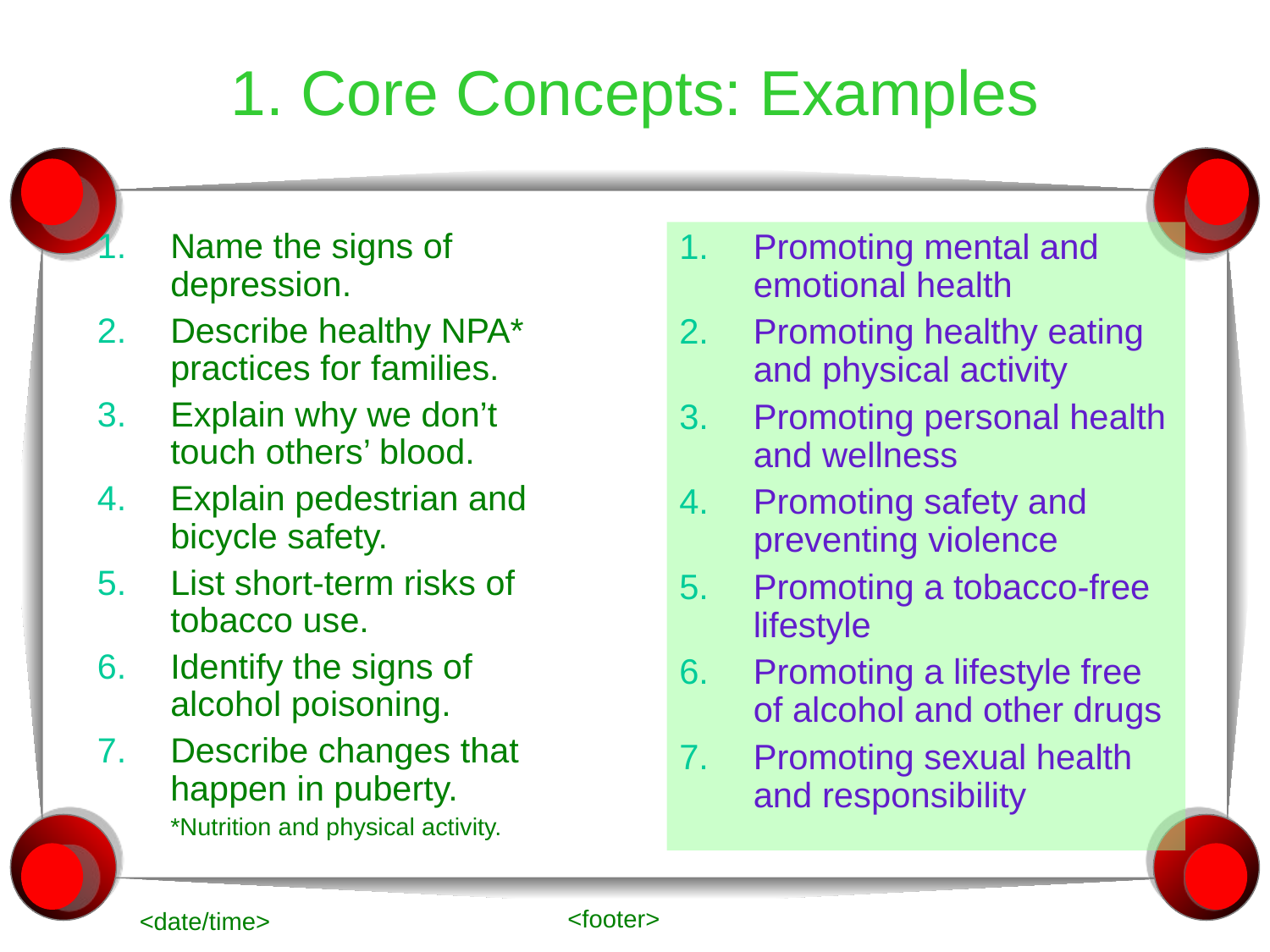

# 1. Core Concepts: Examples
Name the signs of depression.
Describe healthy NPA* practices for families.
Explain why we don’t touch others’ blood.
Explain pedestrian and bicycle safety.
List short-term risks of tobacco use.
Identify the signs of alcohol poisoning.
Describe changes that happen in puberty.
*Nutrition and physical activity.
Promoting mental and emotional health
Promoting healthy eating and physical activity
Promoting personal health and wellness
Promoting safety and preventing violence
Promoting a tobacco-free lifestyle
Promoting a lifestyle free of alcohol and other drugs
Promoting sexual health and responsibility
<footer>
<date/time>

## Slide 14
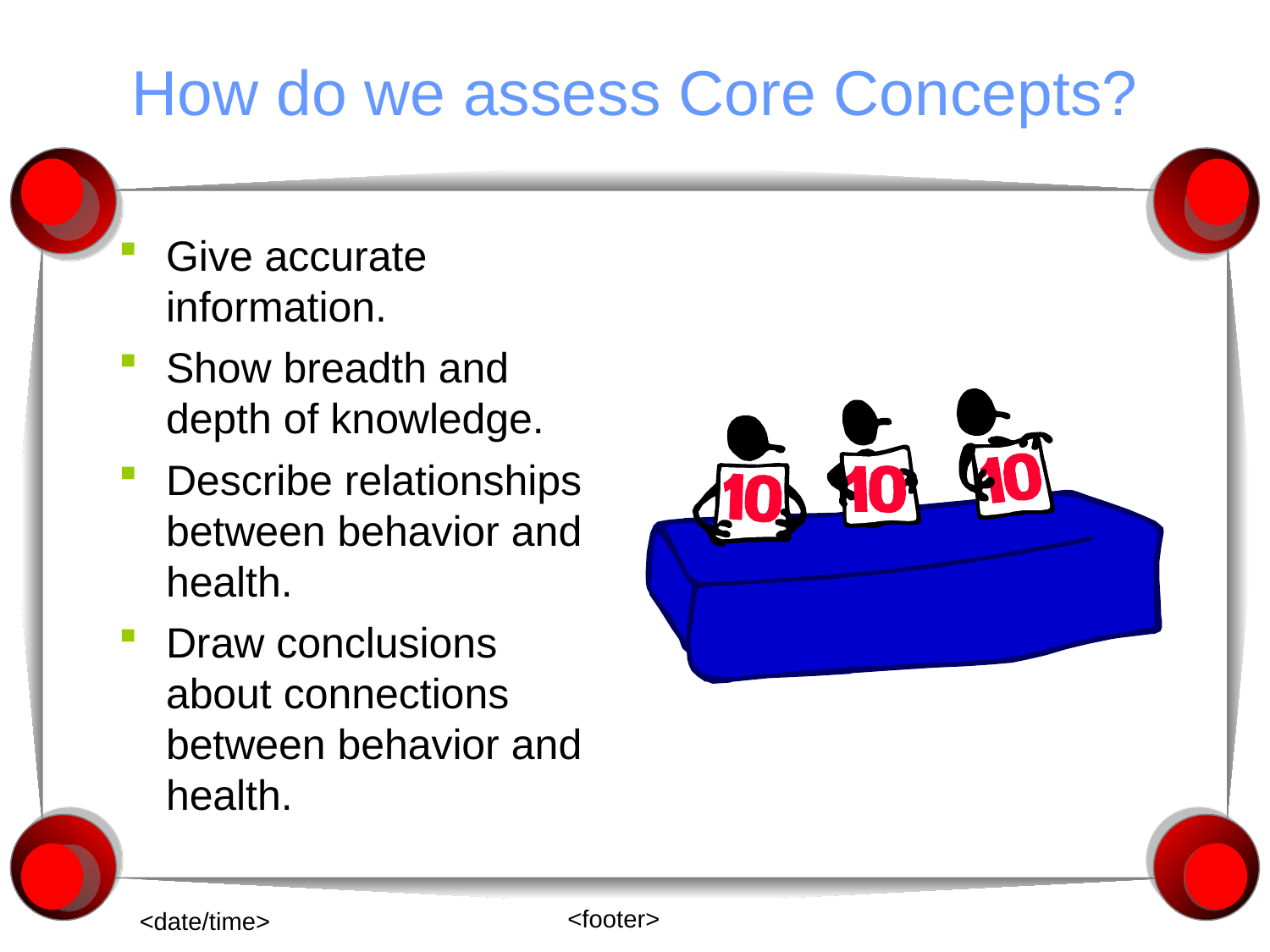

# How do we assess Core Concepts?
Give accurate information.
Show breadth and depth of knowledge.
Describe relationships between behavior and health.
Draw conclusions about connections between behavior and health.
<footer>
<date/time>

## Slide 15
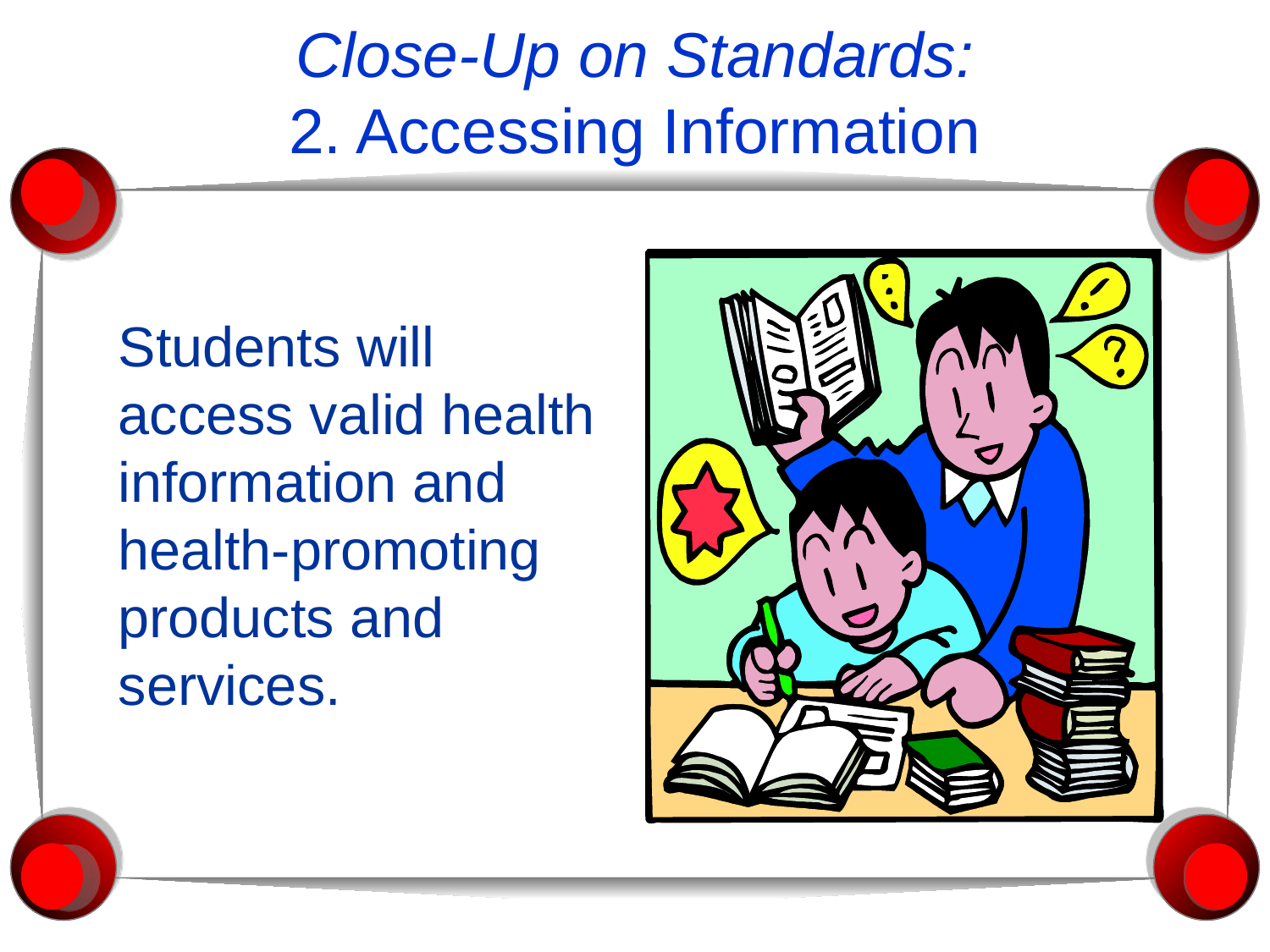

# Close-Up on Standards: 2. Accessing Information
Students will access valid health information and health-promoting products and services.

## Slide 16
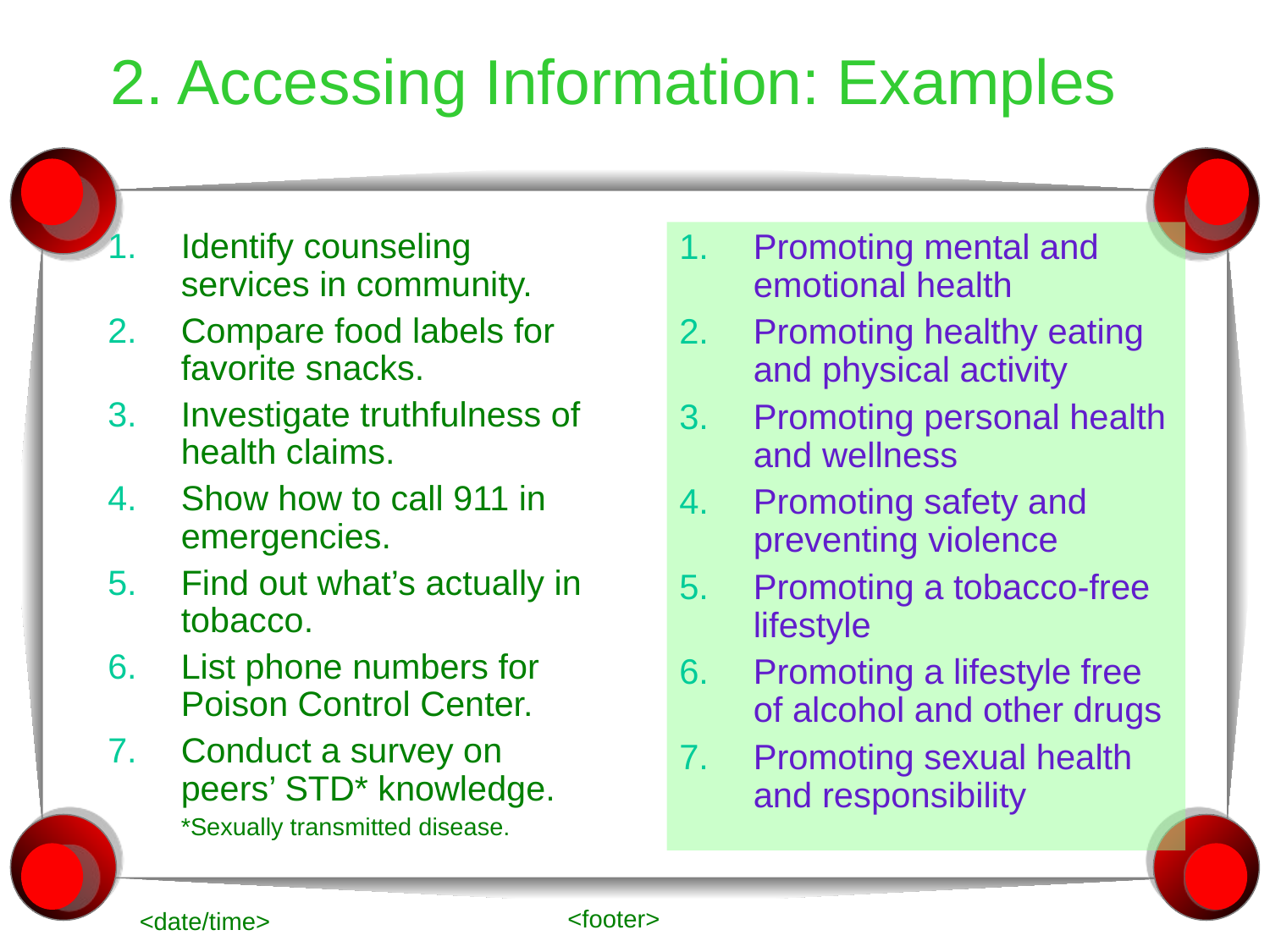

# 2. Accessing Information: Examples
Identify counseling services in community.
Compare food labels for favorite snacks.
Investigate truthfulness of health claims.
Show how to call 911 in emergencies.
Find out what’s actually in tobacco.
List phone numbers for Poison Control Center.
Conduct a survey on peers’ STD* knowledge.
*Sexually transmitted disease.
Promoting mental and emotional health
Promoting healthy eating and physical activity
Promoting personal health and wellness
Promoting safety and preventing violence
Promoting a tobacco-free lifestyle
Promoting a lifestyle free of alcohol and other drugs
Promoting sexual health and responsibility
<footer>
<date/time>

## Slide 17
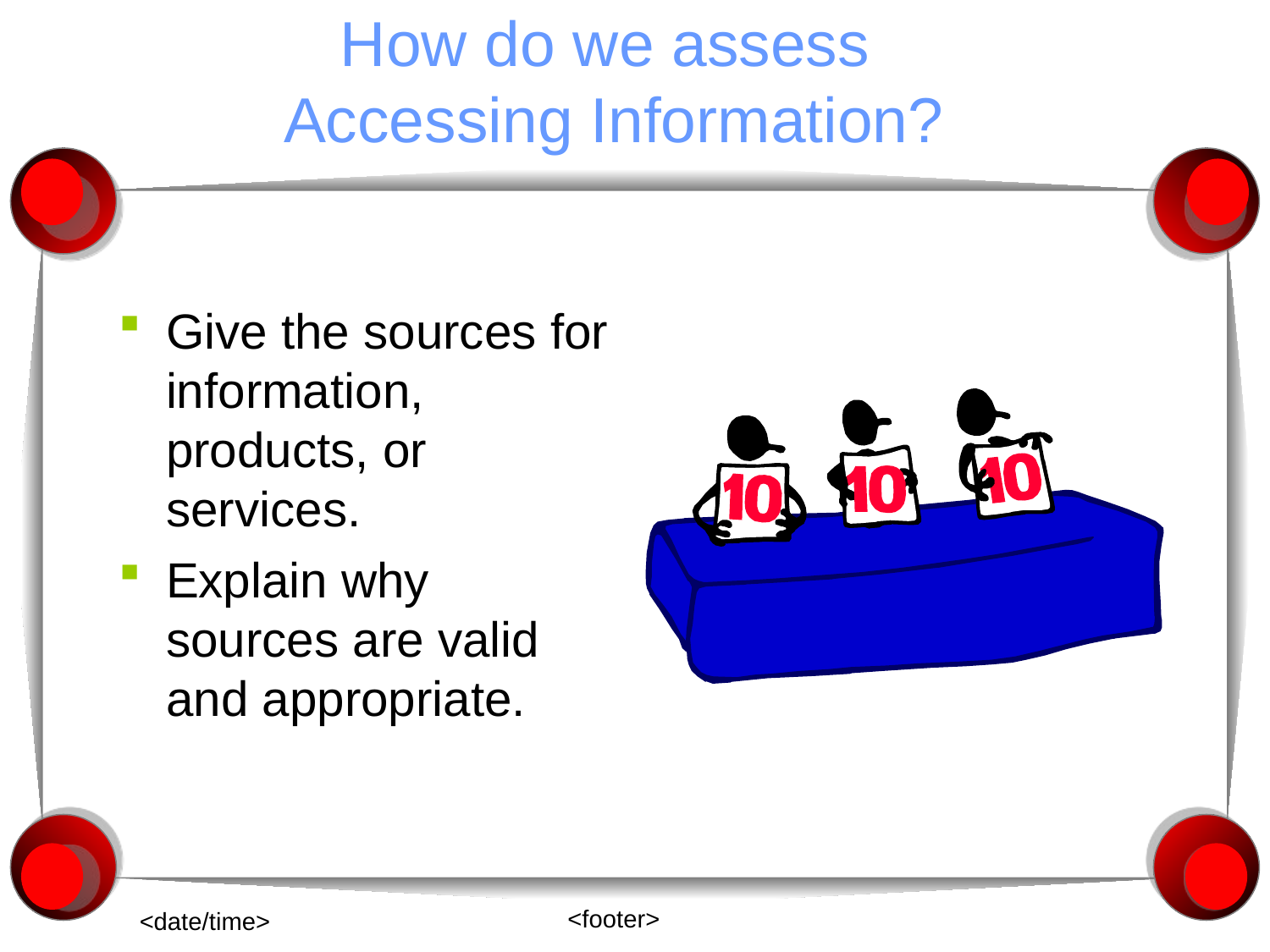

# How do we assess Accessing Information?
Give the sources for information, products, or services.
Explain why sources are valid and appropriate.
<footer>
<date/time>

## Slide 18
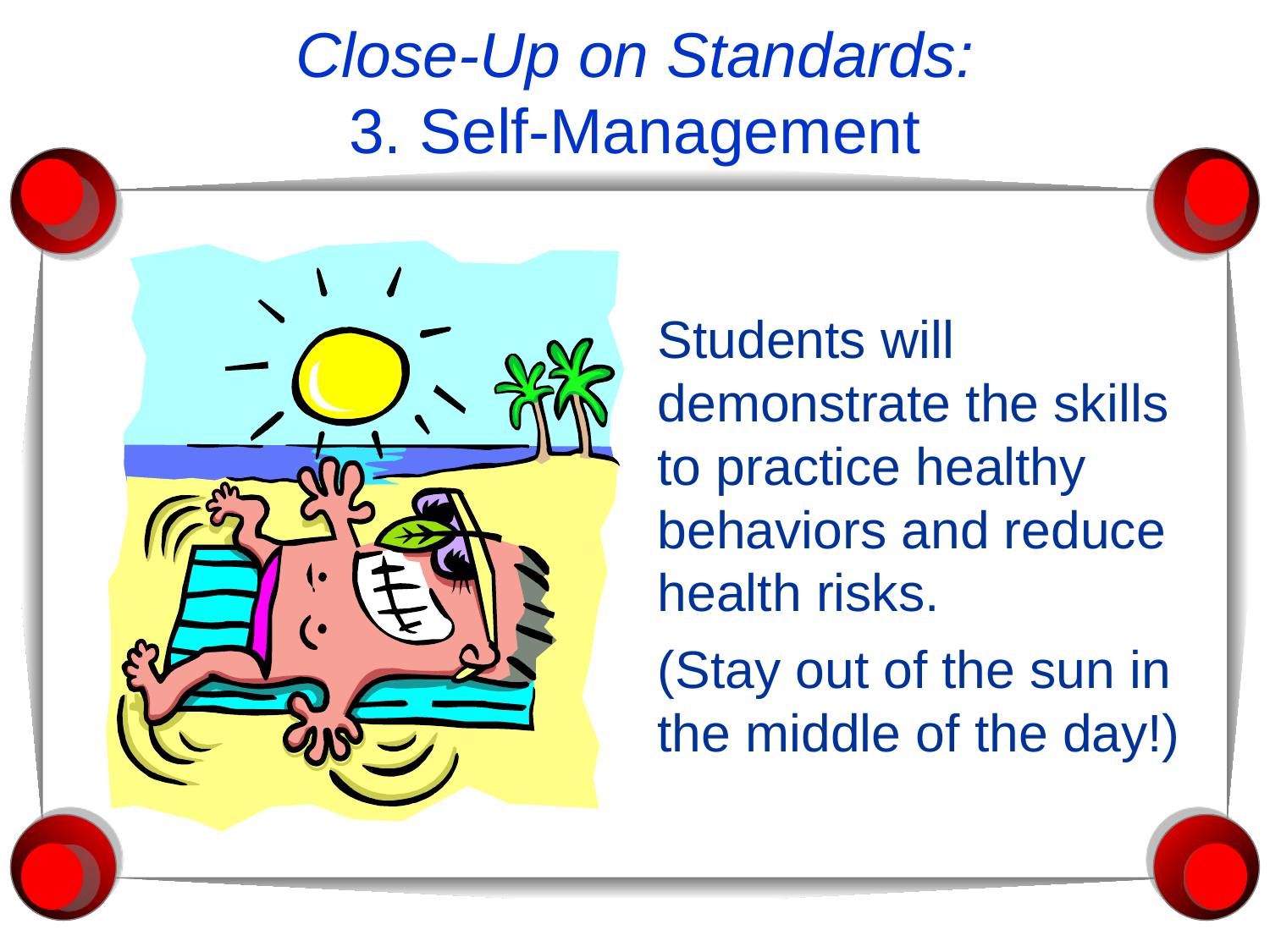

# Close-Up on Standards: 3. Self-Management
Students will demonstrate the skills to practice healthy behaviors and reduce health risks.
(Stay out of the sun in the middle of the day!)

## Slide 19
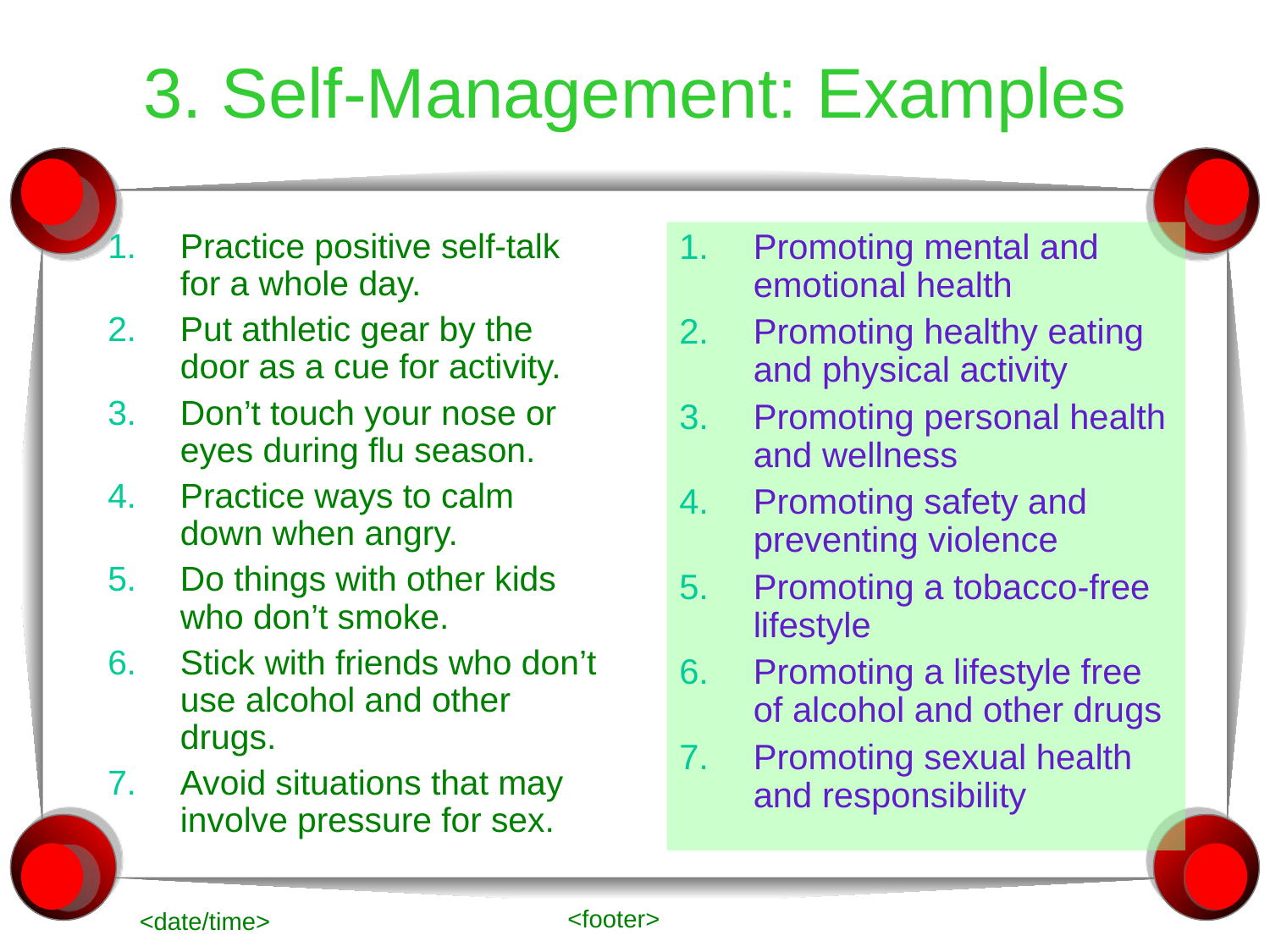

# 3. Self-Management: Examples
Practice positive self-talk for a whole day.
Put athletic gear by the door as a cue for activity.
Don’t touch your nose or eyes during flu season.
Practice ways to calm down when angry.
Do things with other kids who don’t smoke.
Stick with friends who don’t use alcohol and other drugs.
Avoid situations that may involve pressure for sex.
Promoting mental and emotional health
Promoting healthy eating and physical activity
Promoting personal health and wellness
Promoting safety and preventing violence
Promoting a tobacco-free lifestyle
Promoting a lifestyle free of alcohol and other drugs
Promoting sexual health and responsibility
<footer>
<date/time>

## Slide 20
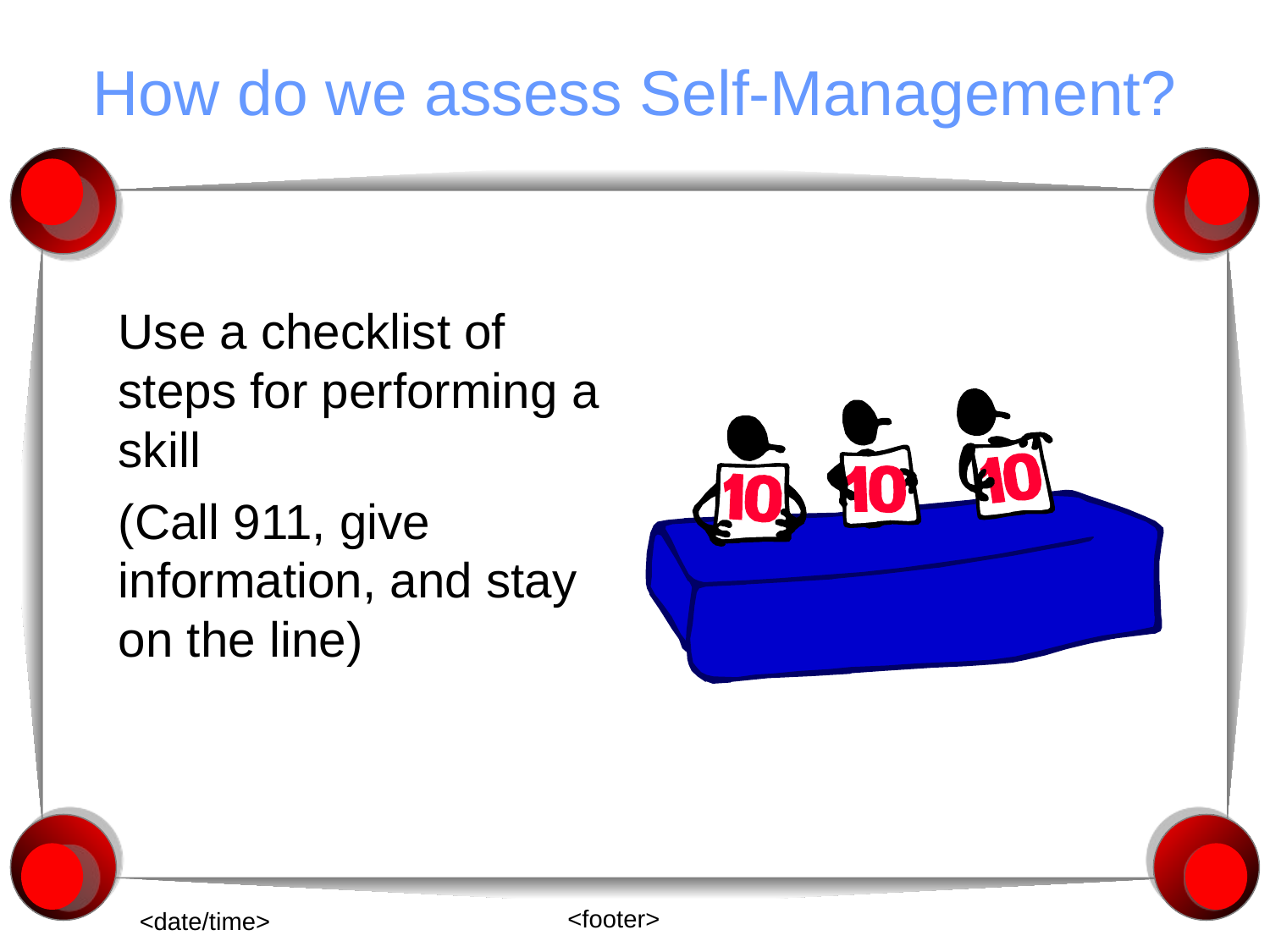

# How do we assess Self-Management?
Use a checklist of steps for performing a skill
(Call 911, give information, and stay on the line)
<footer>
<date/time>

## Slide 21
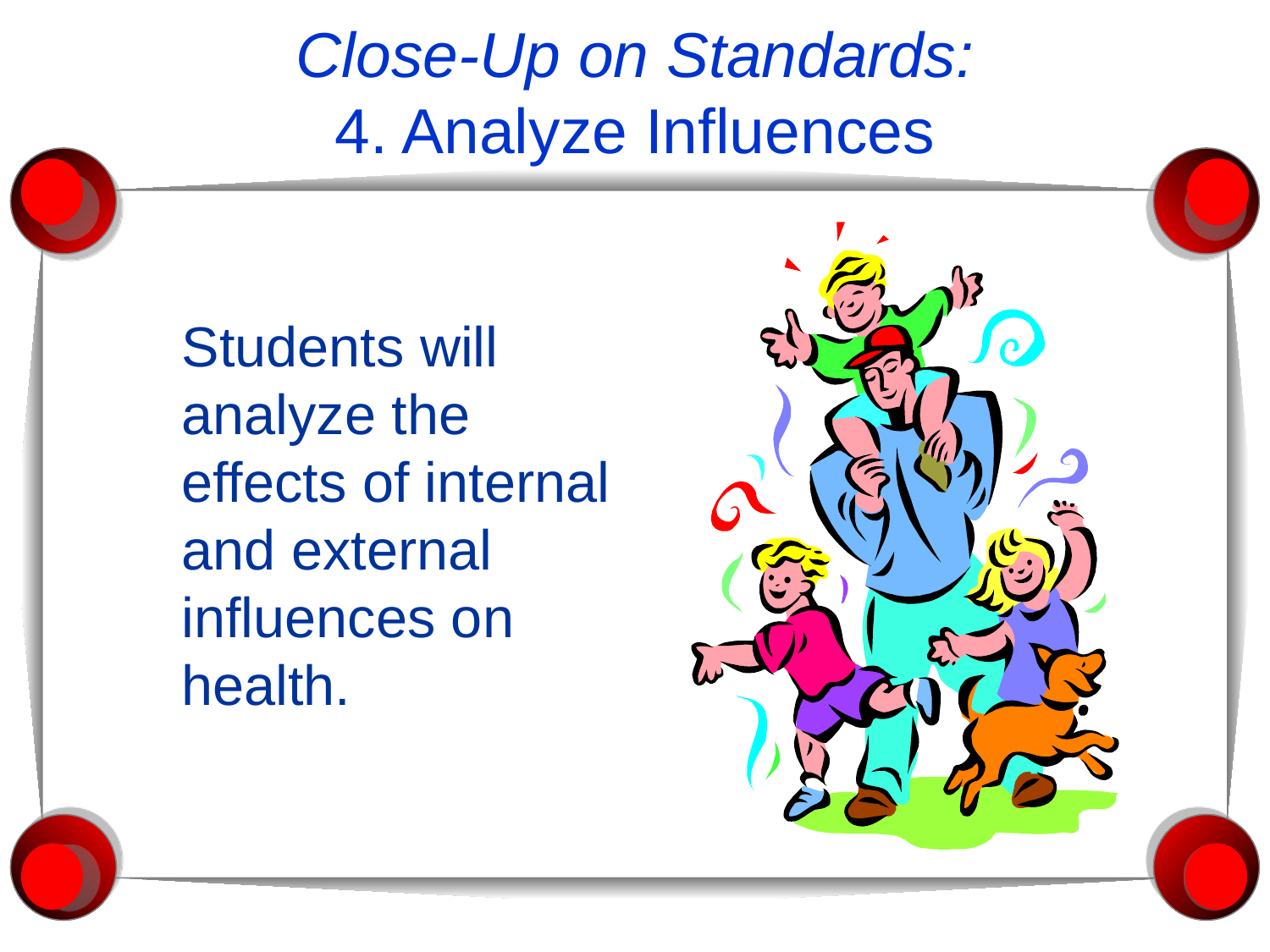

# Close-Up on Standards: 4. Analyze Influences
Students will analyze the effects of internal and external influences on health.

## Slide 22
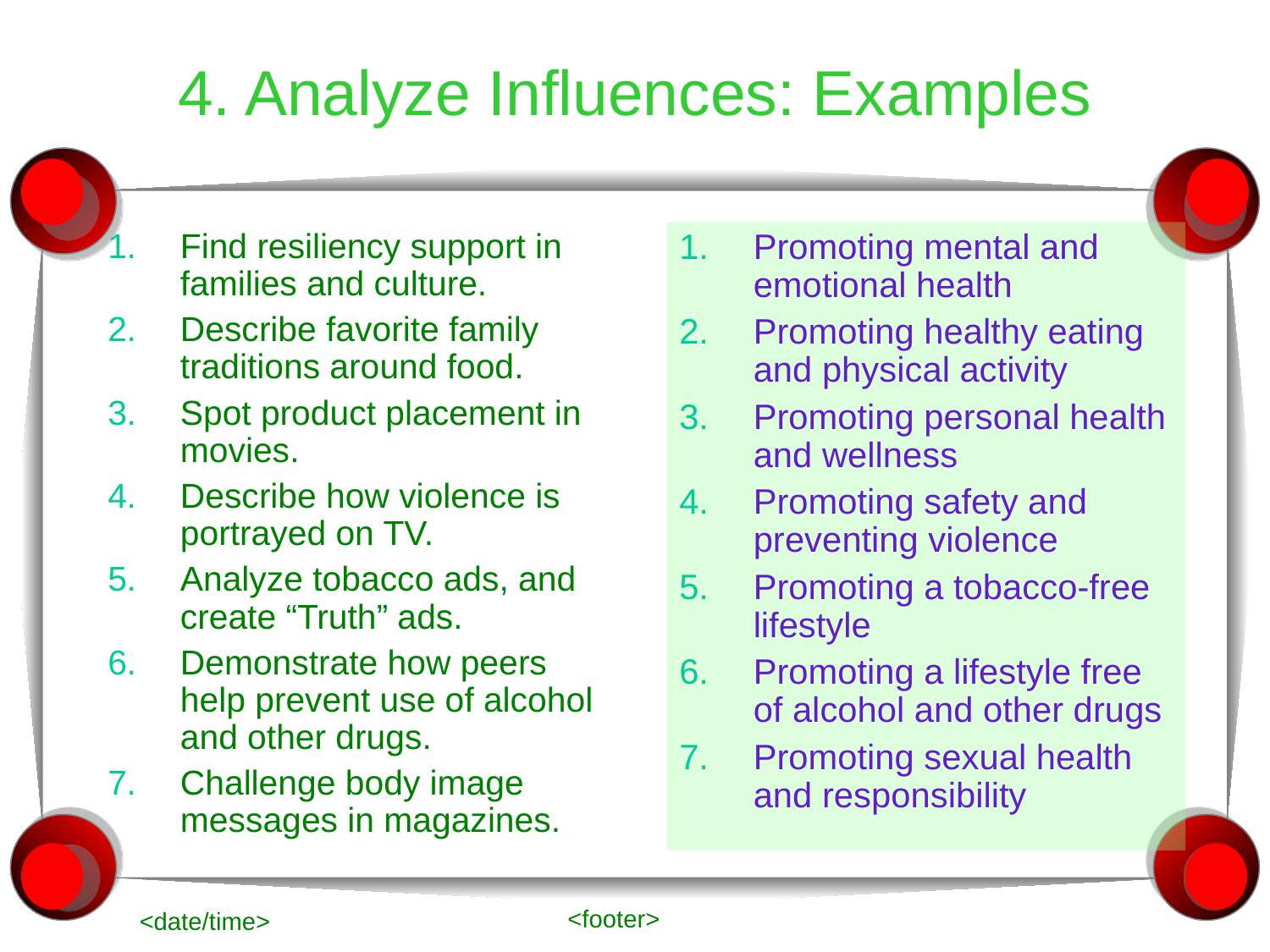

# 4. Analyze Influences: Examples
Find resiliency support in families and culture.
Describe favorite family traditions around food.
Spot product placement in movies.
Describe how violence is portrayed on TV.
Analyze tobacco ads, and create “Truth” ads.
Demonstrate how peers help prevent use of alcohol and other drugs.
Challenge body image messages in magazines.
Promoting mental and emotional health
Promoting healthy eating and physical activity
Promoting personal health and wellness
Promoting safety and preventing violence
Promoting a tobacco-free lifestyle
Promoting a lifestyle free of alcohol and other drugs
Promoting sexual health and responsibility
<footer>
<date/time>

## Slide 23
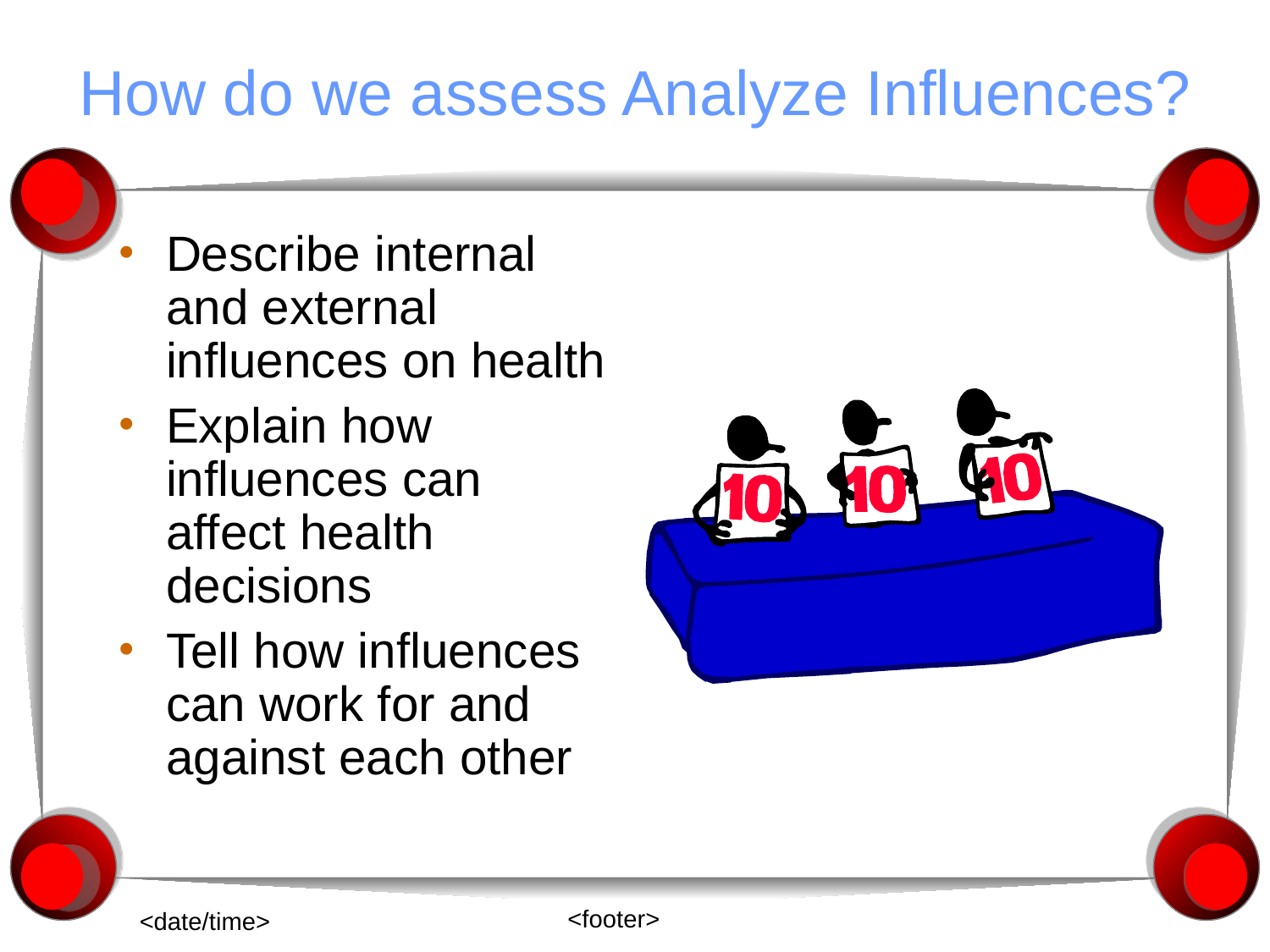

# How do we assess Analyze Influences?
Describe internal and external influences on health
Explain how influences can affect health decisions
Tell how influences can work for and against each other
<footer>
<date/time>

## Slide 24
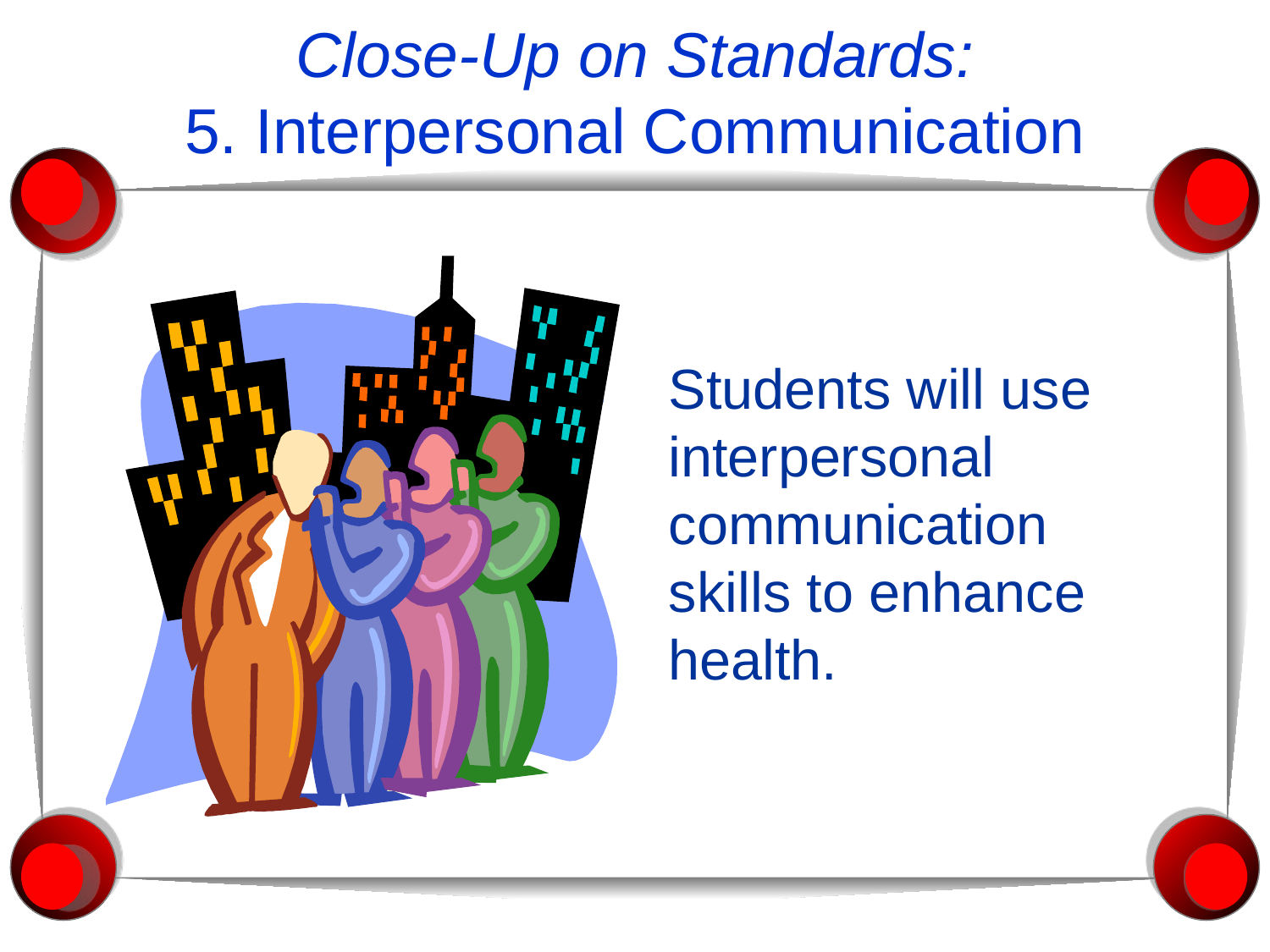

# Close-Up on Standards: 5. Interpersonal Communication
Students will use interpersonal communication skills to enhance health.

## Slide 25
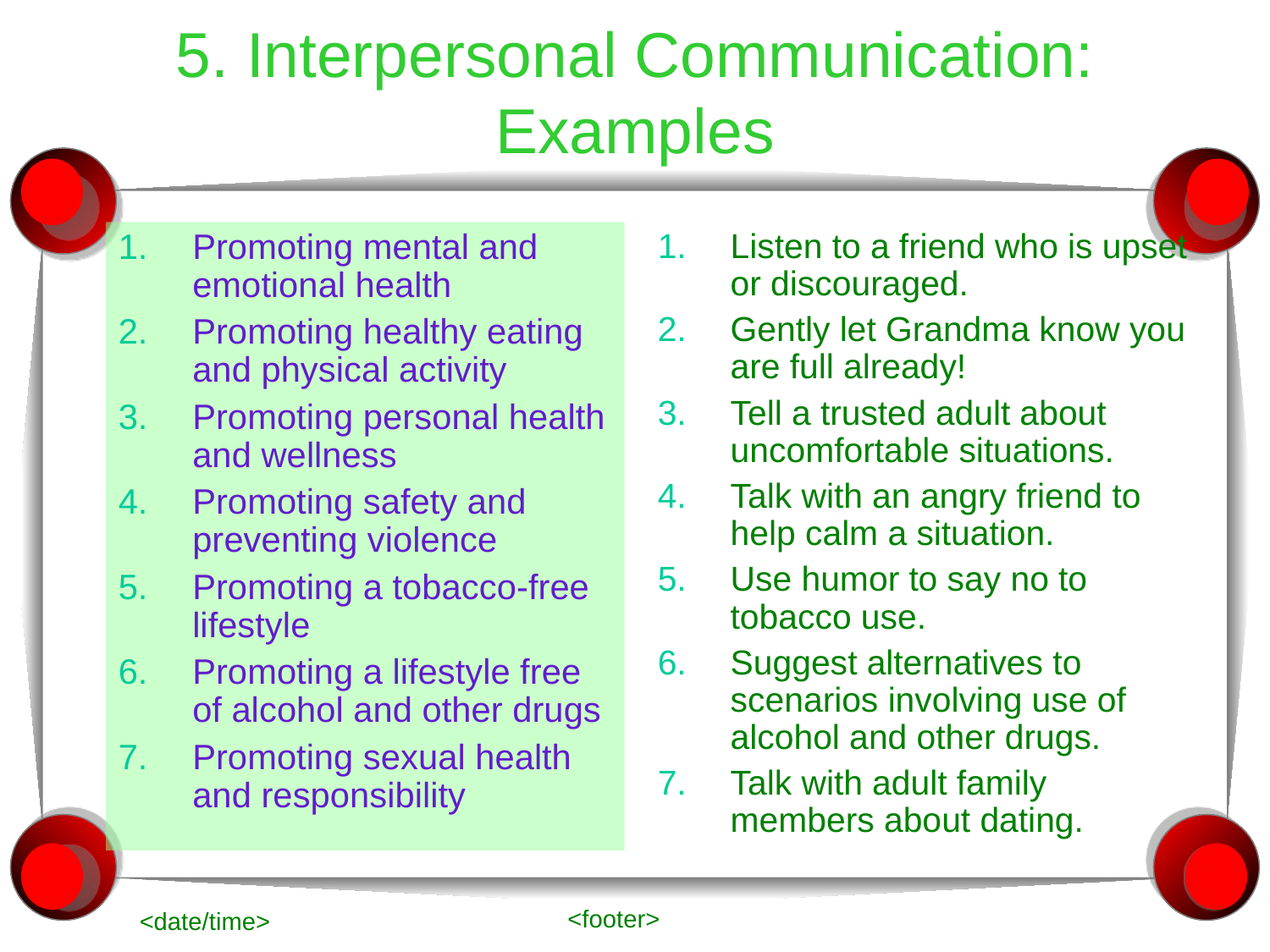

# 5. Interpersonal Communication: Examples
Promoting mental and emotional health
Promoting healthy eating and physical activity
Promoting personal health and wellness
Promoting safety and preventing violence
Promoting a tobacco-free lifestyle
Promoting a lifestyle free of alcohol and other drugs
Promoting sexual health and responsibility
Listen to a friend who is upset or discouraged.
Gently let Grandma know you are full already!
Tell a trusted adult about uncomfortable situations.
Talk with an angry friend to help calm a situation.
Use humor to say no to tobacco use.
Suggest alternatives to scenarios involving use of alcohol and other drugs.
Talk with adult family members about dating.
<footer>
<date/time>

## Slide 26
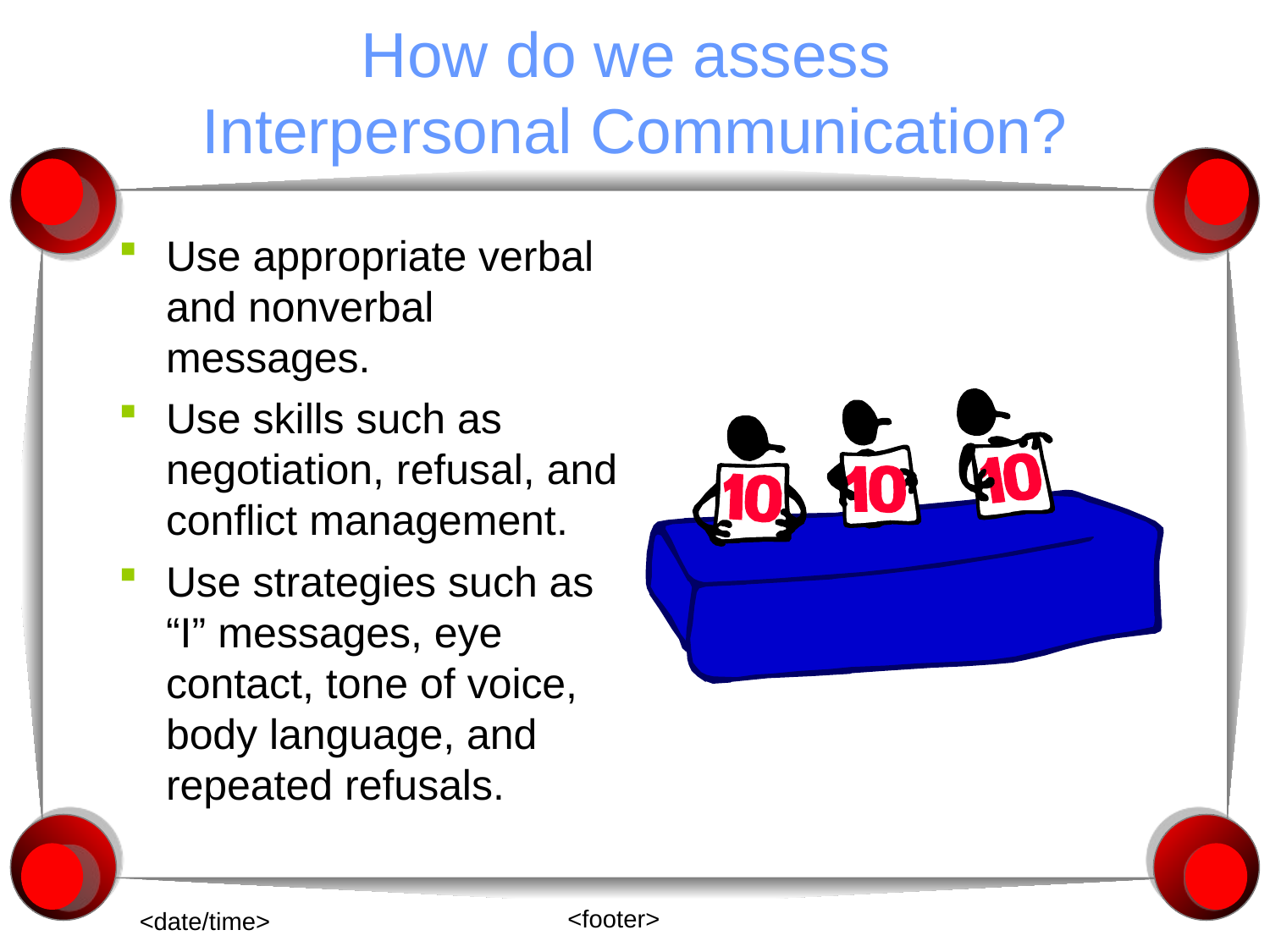

# How do we assess Interpersonal Communication?
Use appropriate verbal and nonverbal messages.
Use skills such as negotiation, refusal, and conflict management.
Use strategies such as “I” messages, eye contact, tone of voice, body language, and repeated refusals.
<footer>
<date/time>

## Slide 27
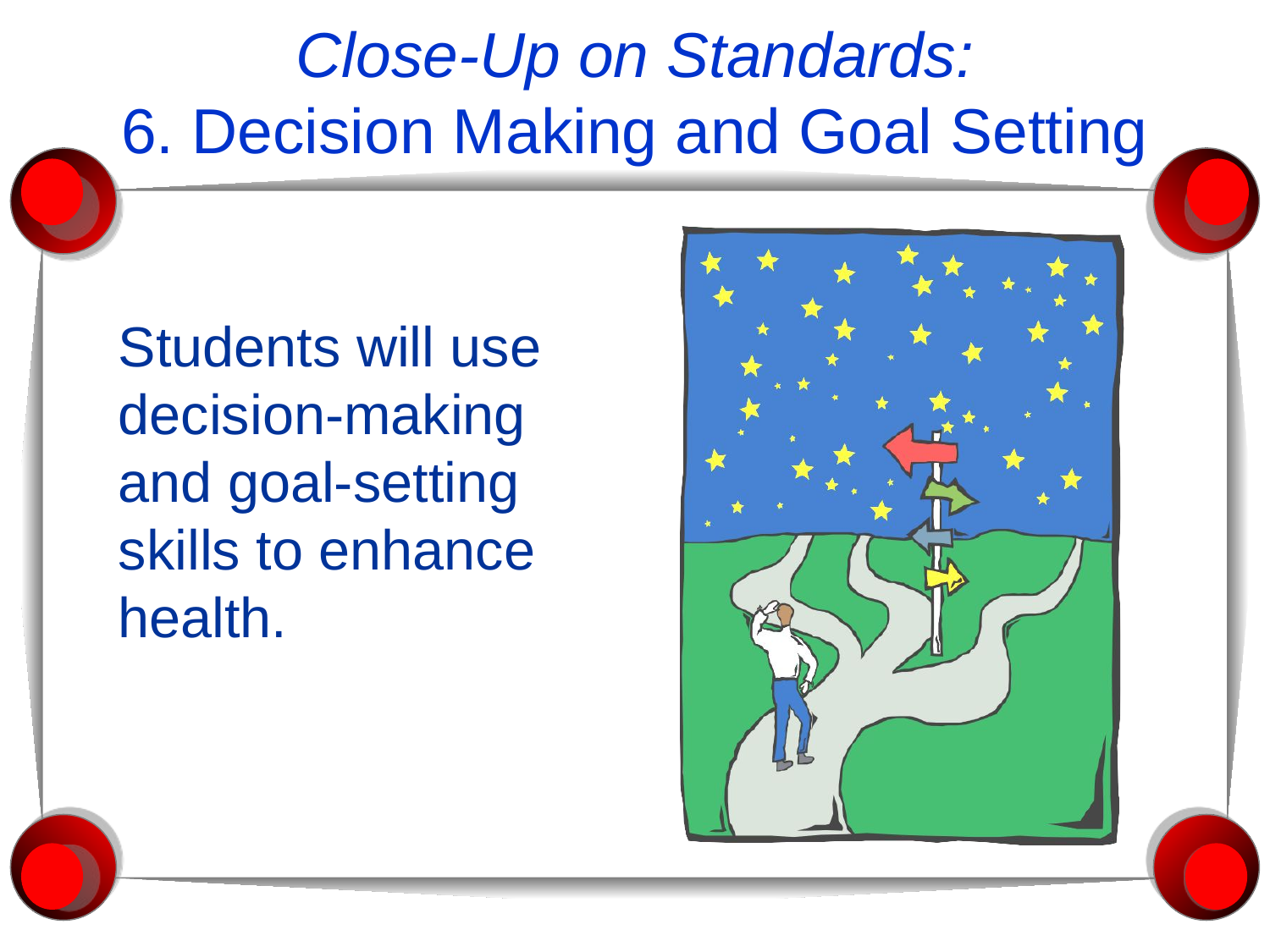

# Close-Up on Standards: 6. Decision Making and Goal Setting
Students will use decision-making and goal-setting skills to enhance health.

## Slide 28
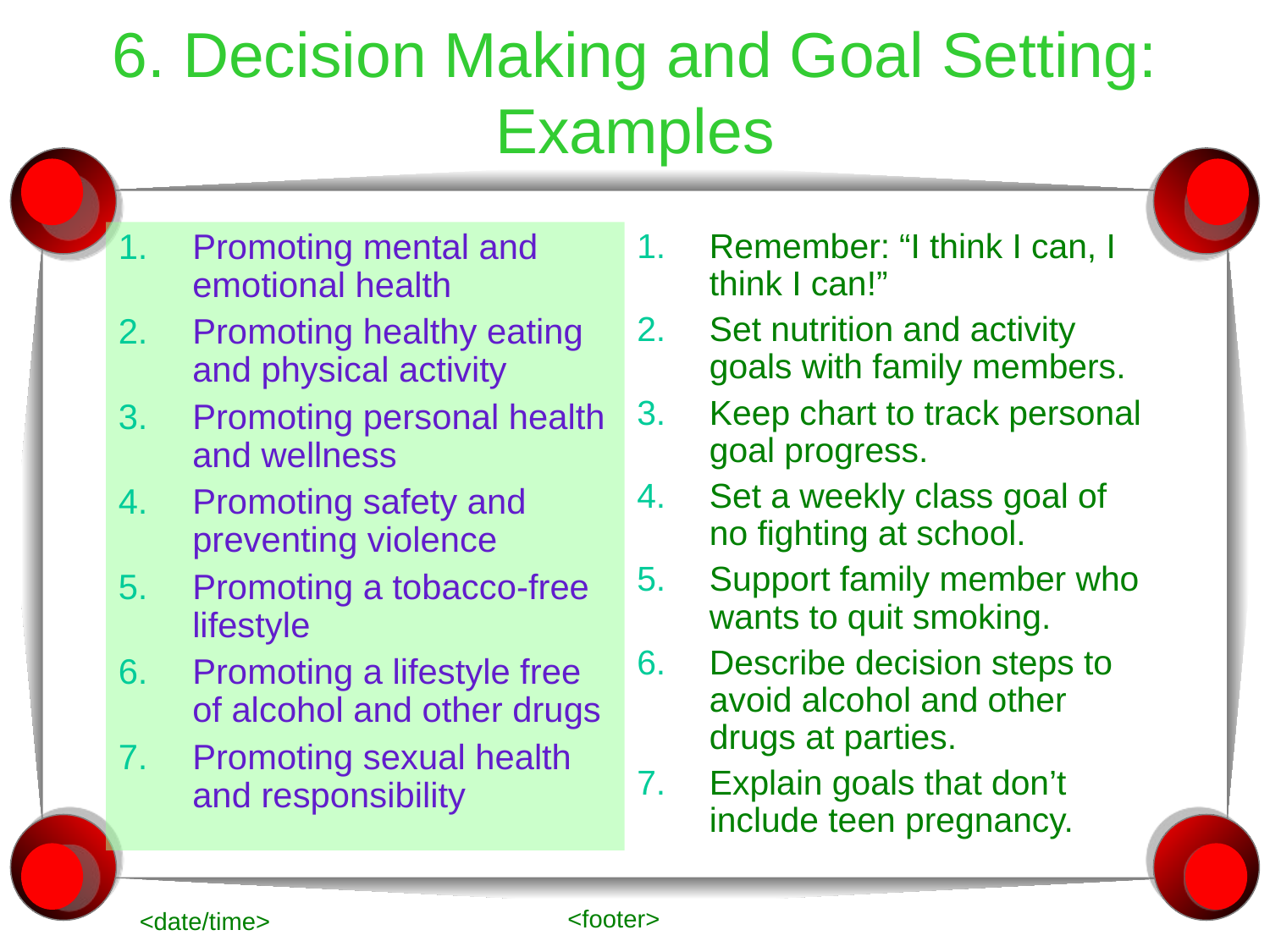

# 6. Decision Making and Goal Setting: Examples
Promoting mental and emotional health
Promoting healthy eating and physical activity
Promoting personal health and wellness
Promoting safety and preventing violence
Promoting a tobacco-free lifestyle
Promoting a lifestyle free of alcohol and other drugs
Promoting sexual health and responsibility
Remember: “I think I can, I think I can!”
Set nutrition and activity goals with family members.
Keep chart to track personal goal progress.
Set a weekly class goal of no fighting at school.
Support family member who wants to quit smoking.
Describe decision steps to avoid alcohol and other drugs at parties.
Explain goals that don’t include teen pregnancy.
<footer>
<date/time>

## Slide 29
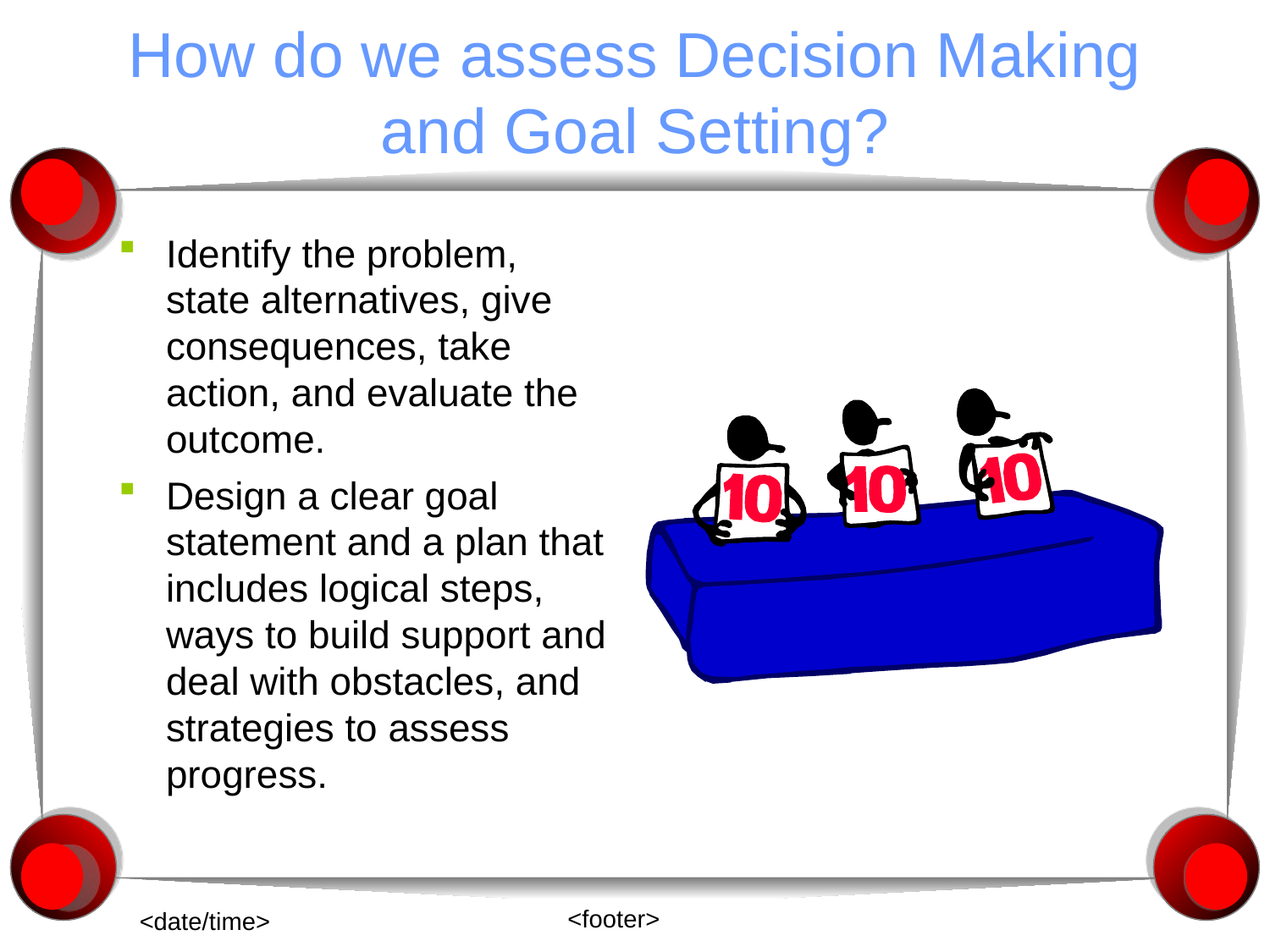

# How do we assess Decision Making and Goal Setting?
Identify the problem, state alternatives, give consequences, take action, and evaluate the outcome.
Design a clear goal statement and a plan that includes logical steps, ways to build support and deal with obstacles, and strategies to assess progress.
<footer>
<date/time>

## Slide 30
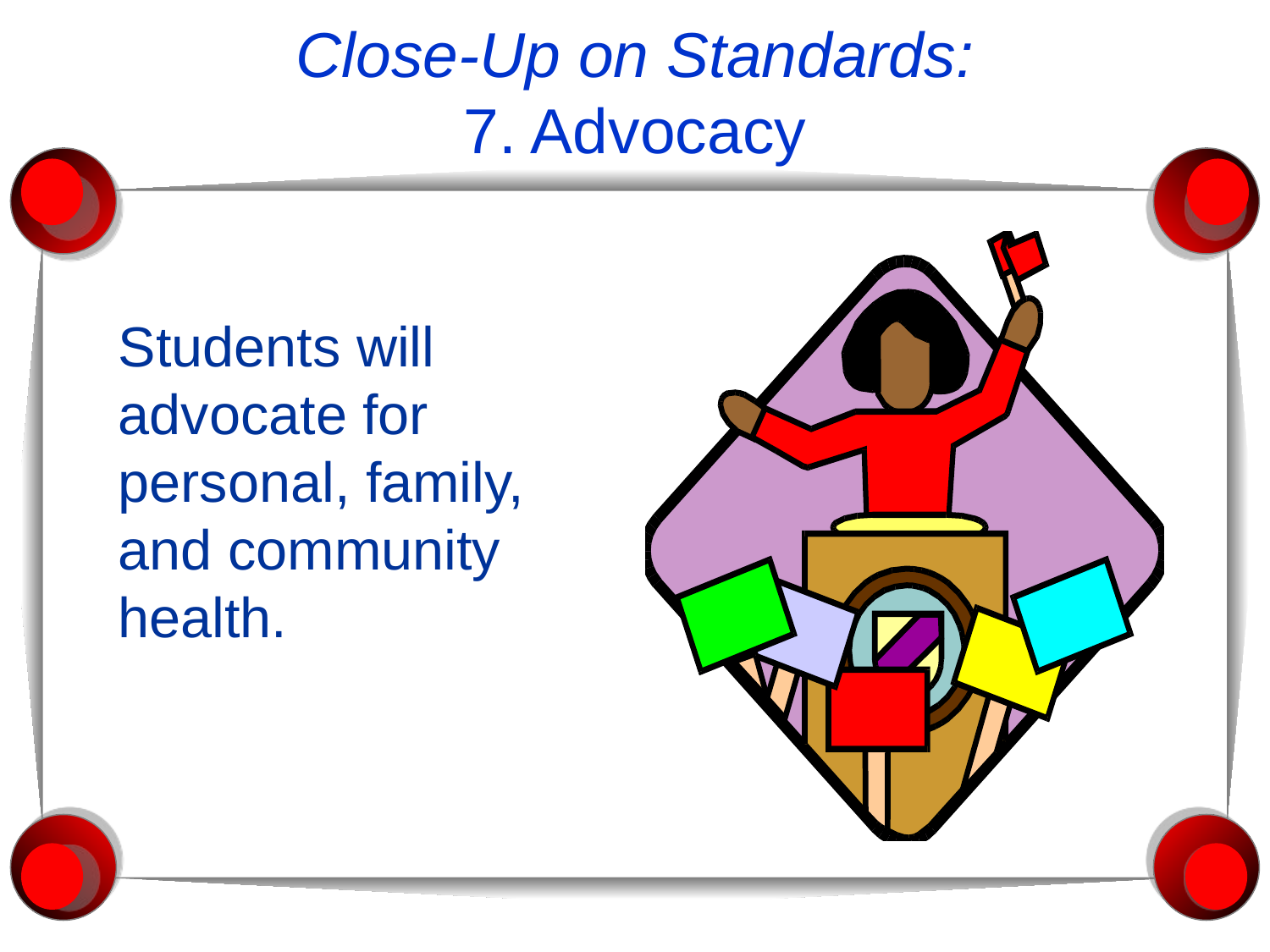

# Close-Up on Standards: 7. Advocacy
Students will advocate for personal, family, and community health.

## Slide 31
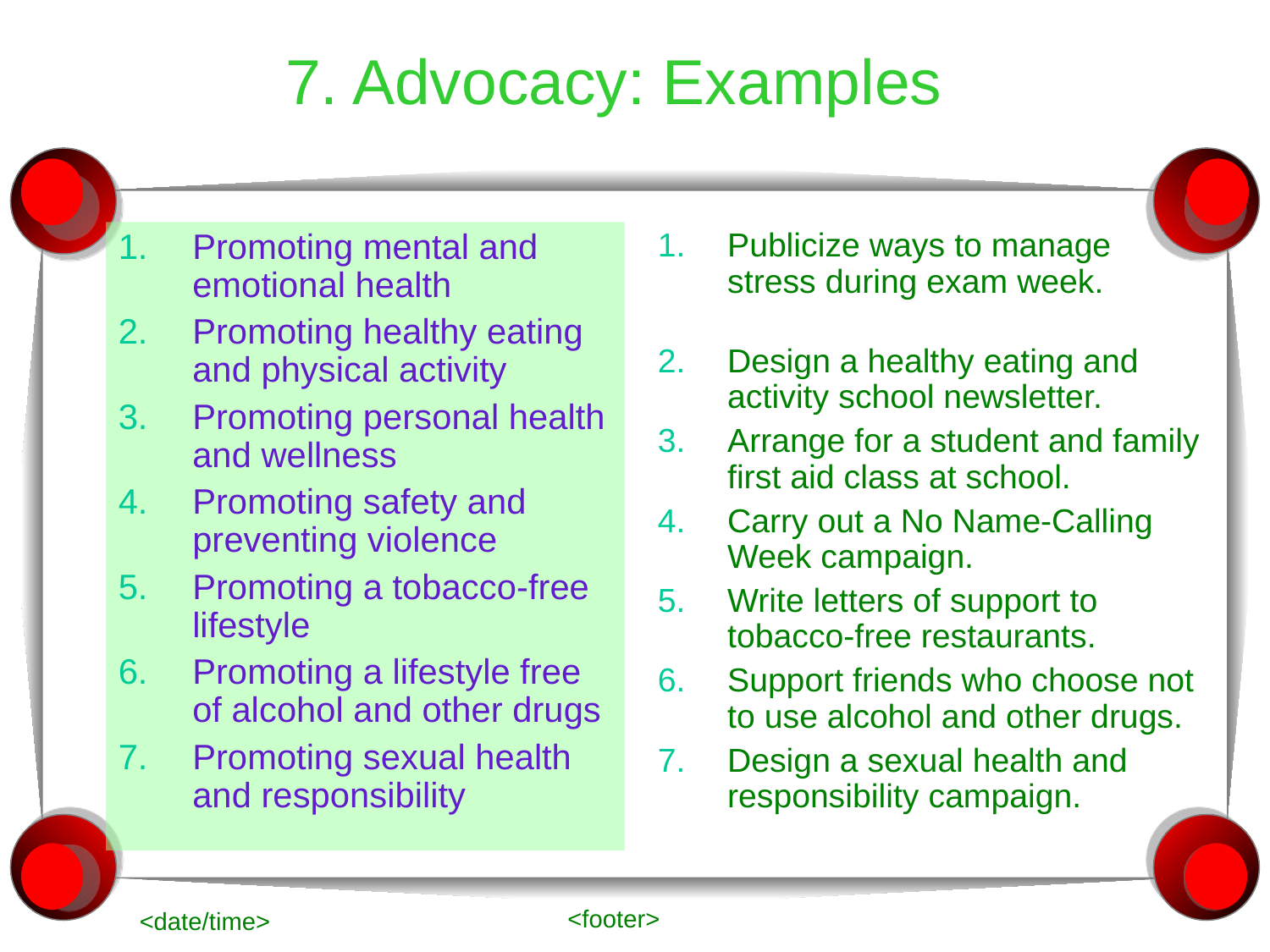

# 7. Advocacy: Examples
Promoting mental and emotional health
Promoting healthy eating and physical activity
Promoting personal health and wellness
Promoting safety and preventing violence
Promoting a tobacco-free lifestyle
Promoting a lifestyle free of alcohol and other drugs
Promoting sexual health and responsibility
Publicize ways to manage stress during exam week.
Design a healthy eating and activity school newsletter.
Arrange for a student and family first aid class at school.
Carry out a No Name-Calling Week campaign.
Write letters of support to tobacco-free restaurants.
Support friends who choose not to use alcohol and other drugs.
Design a sexual health and responsibility campaign.
<footer>
<date/time>

## Slide 32
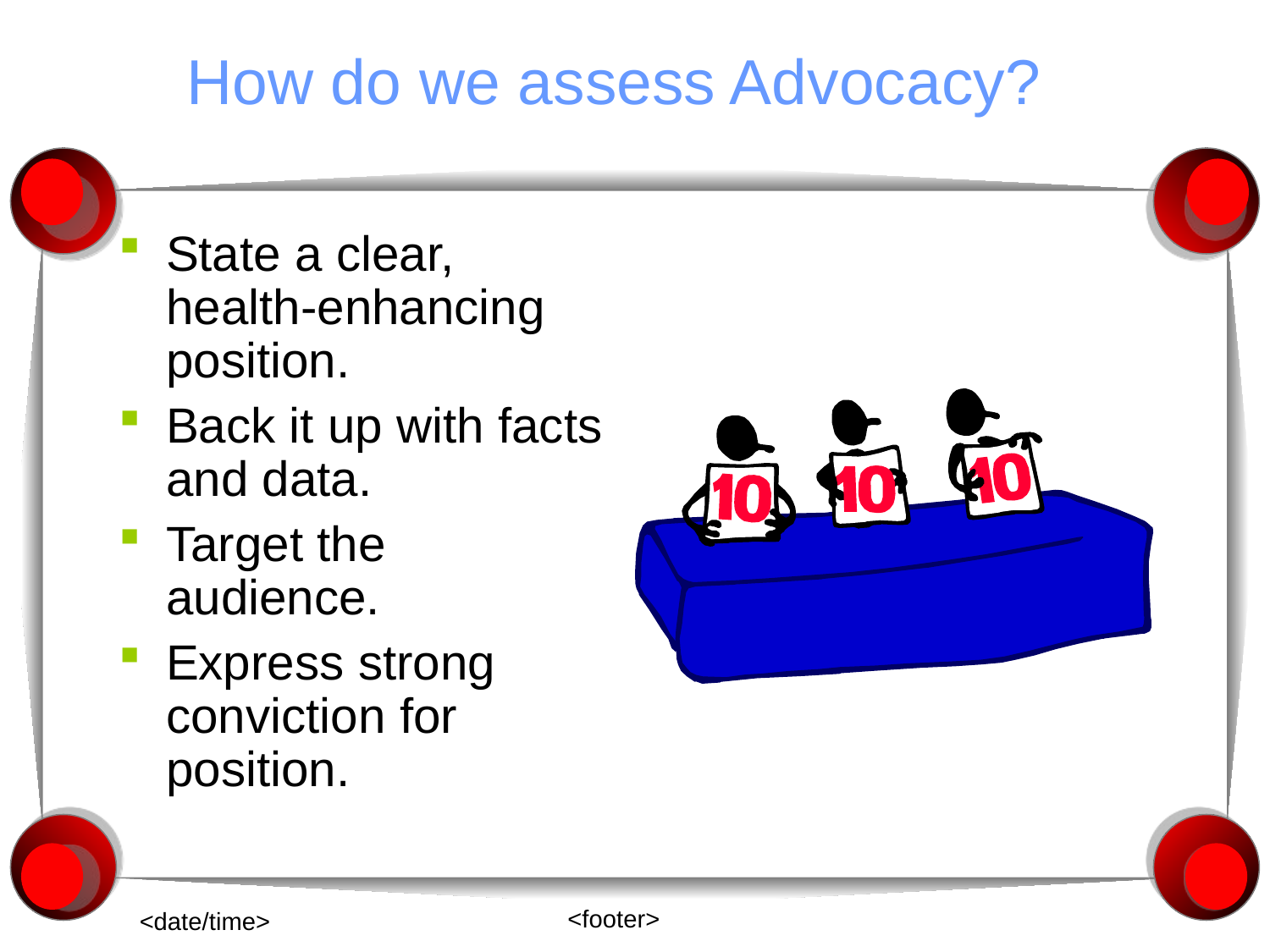

# How do we assess Advocacy?
State a clear, health-enhancing position.
Back it up with facts and data.
Target the audience.
Express strong conviction for position.
<footer>
<date/time>

## Slide 33
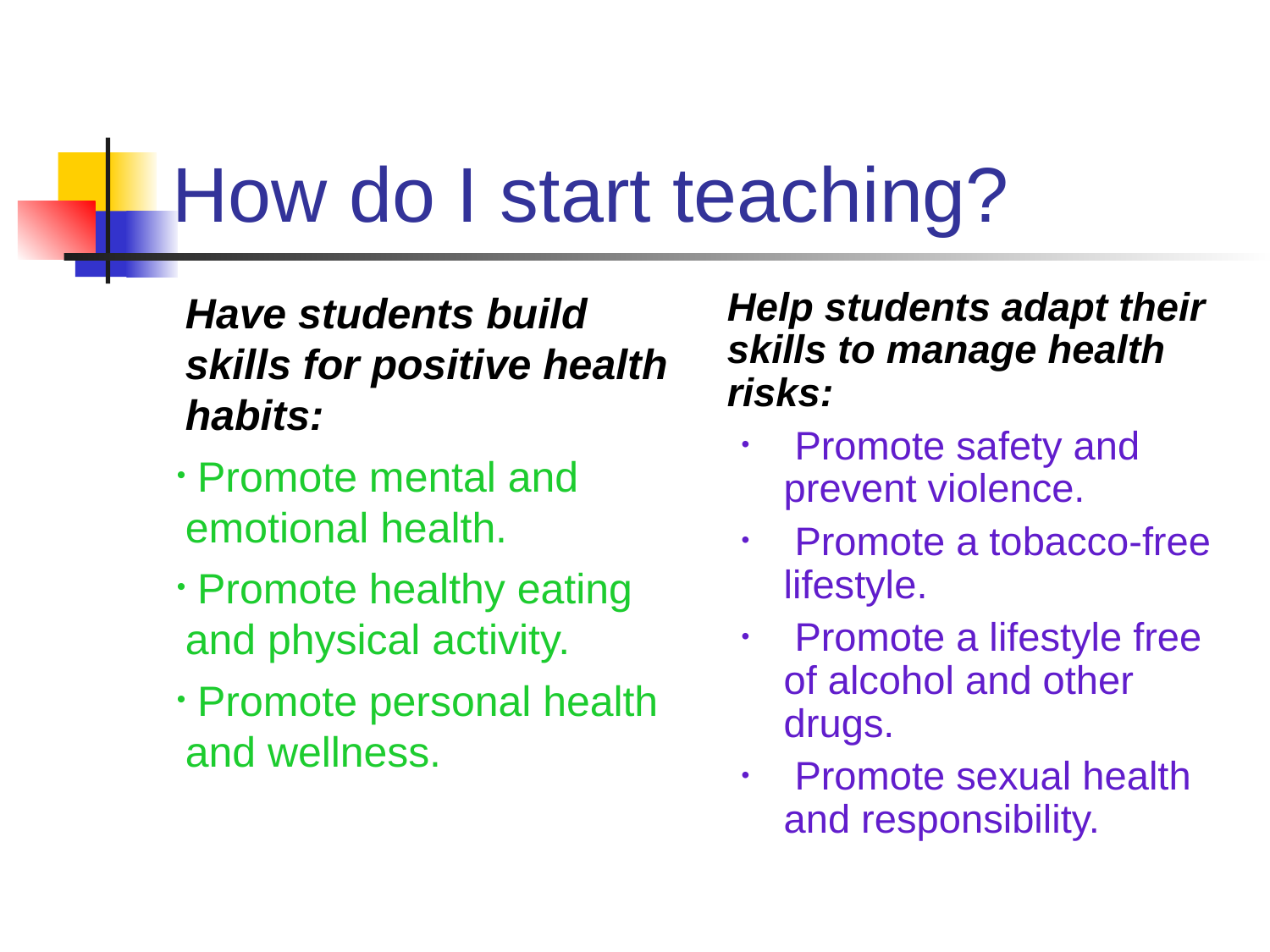

# How do I start teaching?
Have students build skills for positive health habits:
 Promote mental and emotional health.
 Promote healthy eating and physical activity.
 Promote personal health and wellness.
Help students adapt their skills to manage health risks:
 Promote safety and prevent violence.
 Promote a tobacco-free lifestyle.
 Promote a lifestyle free of alcohol and other drugs.
 Promote sexual health and responsibility.

## Slide 34
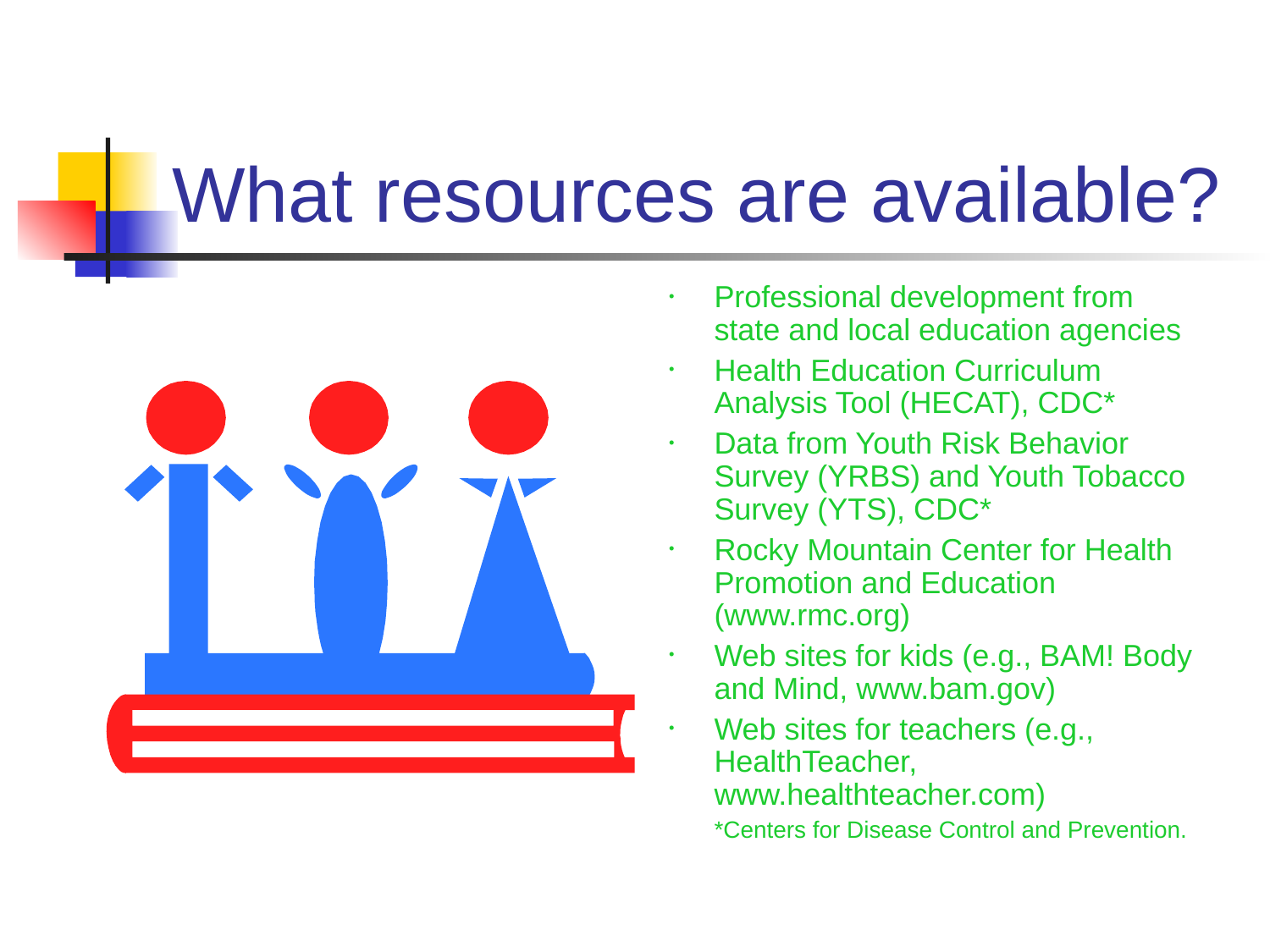

# What resources are available?
Professional development from state and local education agencies
Health Education Curriculum Analysis Tool (HECAT), CDC*
Data from Youth Risk Behavior Survey (YRBS) and Youth Tobacco Survey (YTS), CDC*
Rocky Mountain Center for Health Promotion and Education (www.rmc.org)
Web sites for kids (e.g., BAM! Body and Mind, www.bam.gov)
Web sites for teachers (e.g., HealthTeacher, www.healthteacher.com)
*Centers for Disease Control and Prevention.

## Slide 35
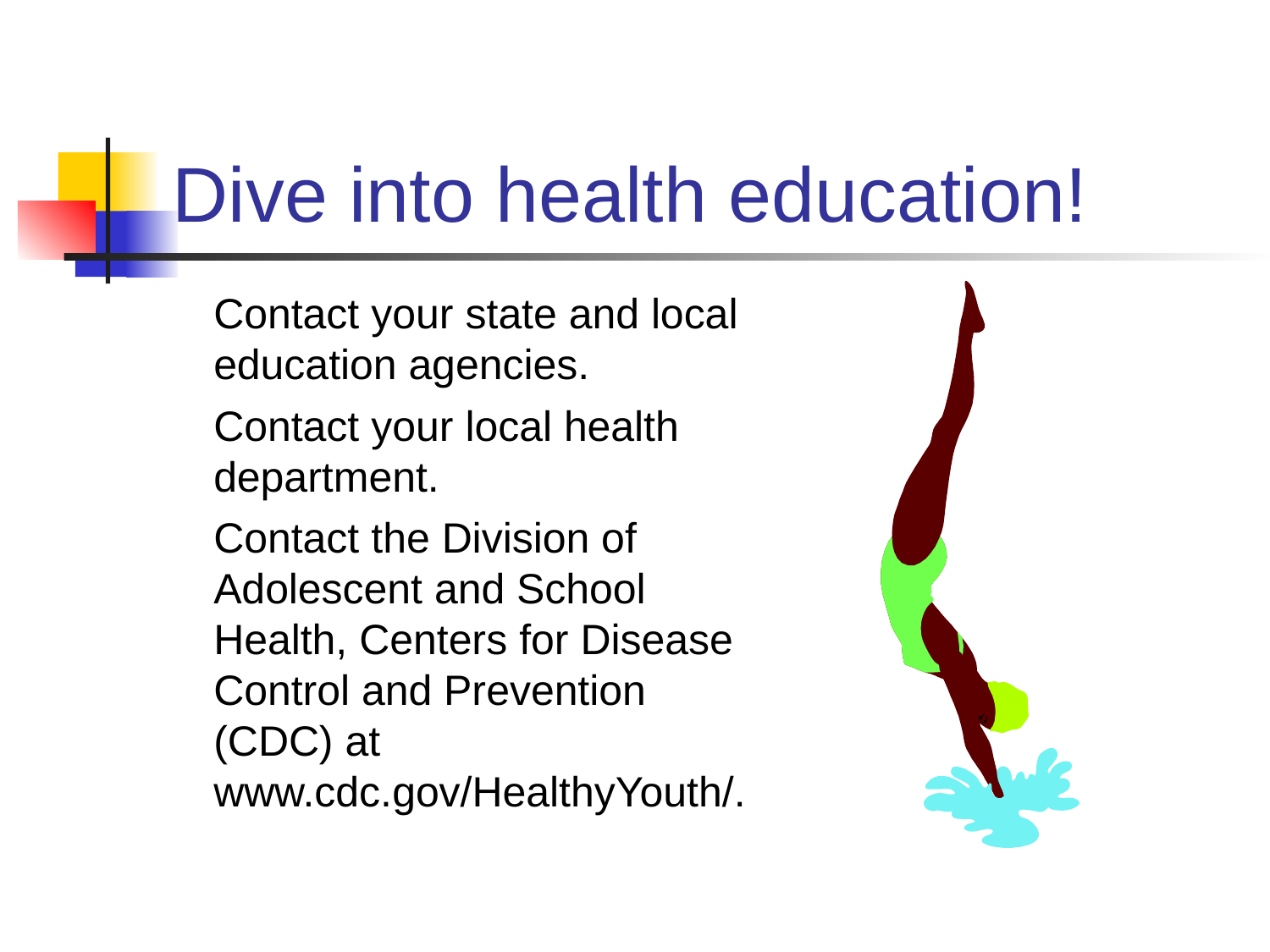

# Dive into health education!
Contact your state and local education agencies.
Contact your local health department.
Contact the Division of Adolescent and School Health, Centers for Disease Control and Prevention (CDC) at www.cdc.gov/HealthyYouth/.

## Slide 36
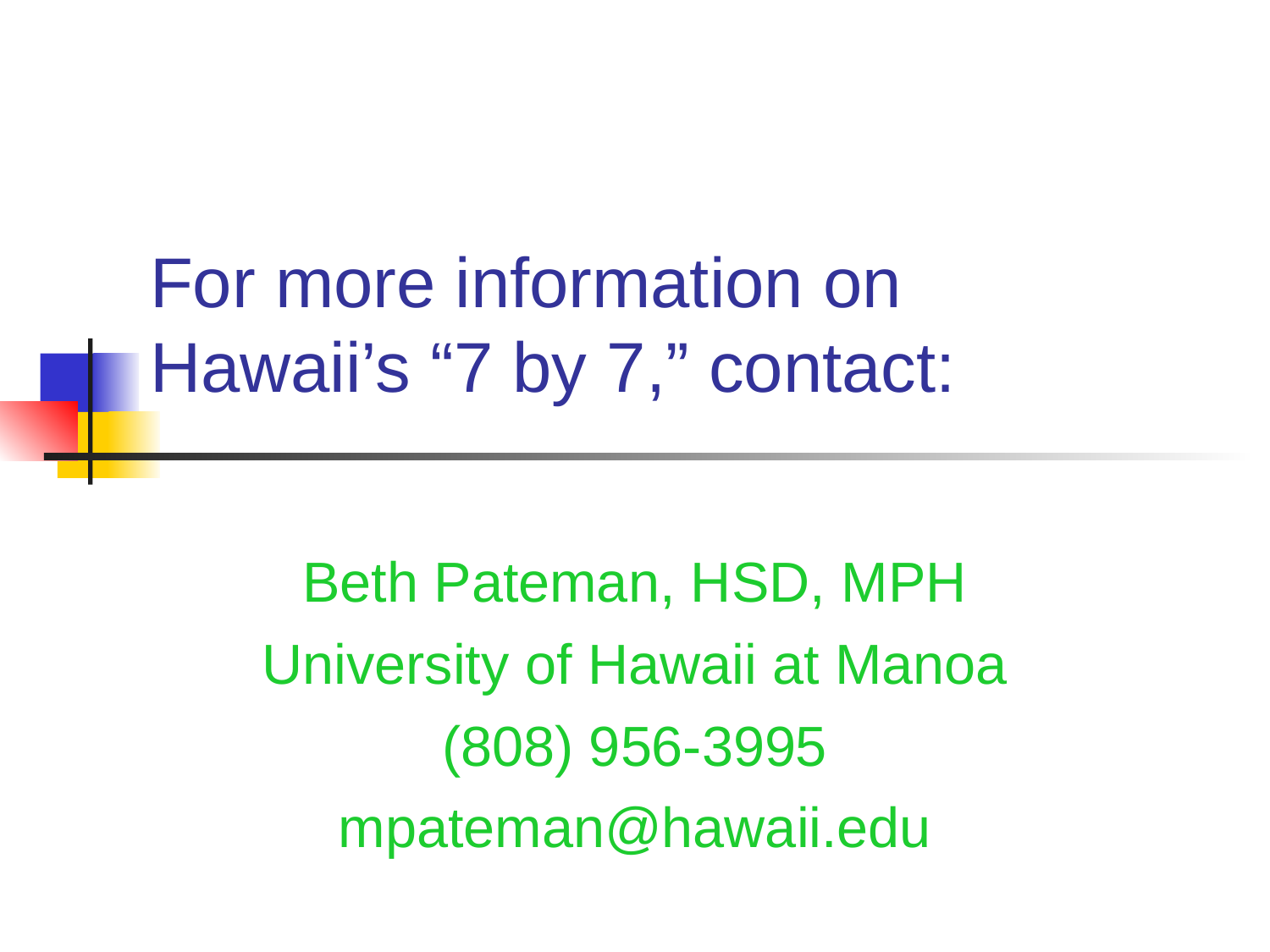

# For more information on Hawaii’s “7 by 7,” contact:
Beth Pateman, HSD, MPH
University of Hawaii at Manoa
(808) 956-3995
mpateman@hawaii.edu
