# Supplementary material for: Hawaii’s “7 by 7” for School Health Education: A PowerPoint Presentation on Integrating the National Health Education Standards With Priority Content Areas for Today’s School Health Education in Grades Kindergarten Through 12
Source: Prev Chronic Dis. 2006 Mar 15;3(2):A63. (PMC1563955)
Supplement: Supplementary file 2 [file 05_0098_01.pdf]

Healthy Keiki, Healthy Hawaii: Hawaii's "7 by 7" for School Health Education

Health Education Standards 101

Beth Pateman, University of Hawaii at Manoa

What was health education like when you were in school?

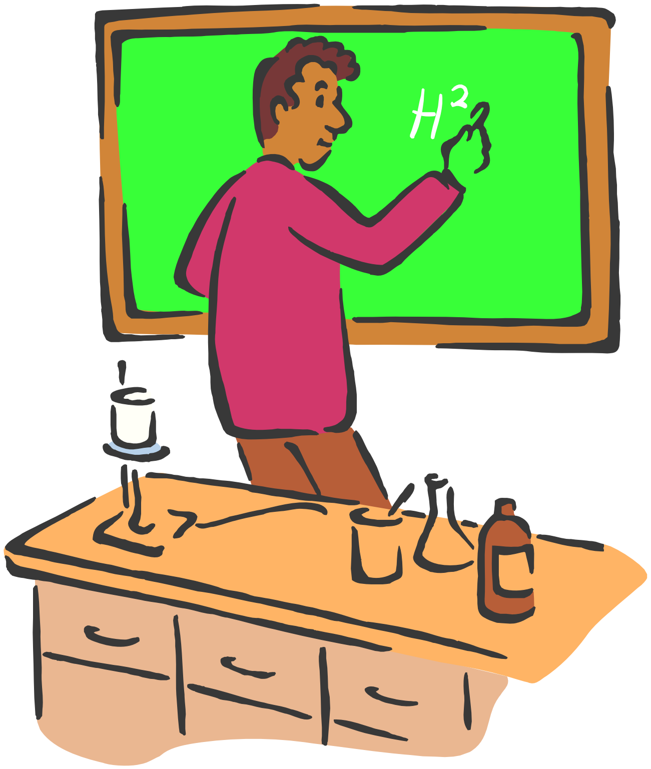

- Dull lectures?
- Outdated textbooks?
- Information only?
- A science course?
- A PE course?
- The same old thing?

Borrrringggg!!!

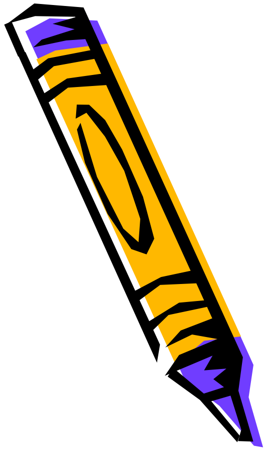

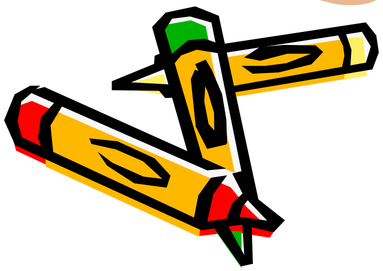

Did health education have anything to do with your real life?

Did it help you...

- Think through real problems and decisions?
- Manage difficult situations?
- Learn how to talk to and get along with others?
- Find out what you **really** needed to know?

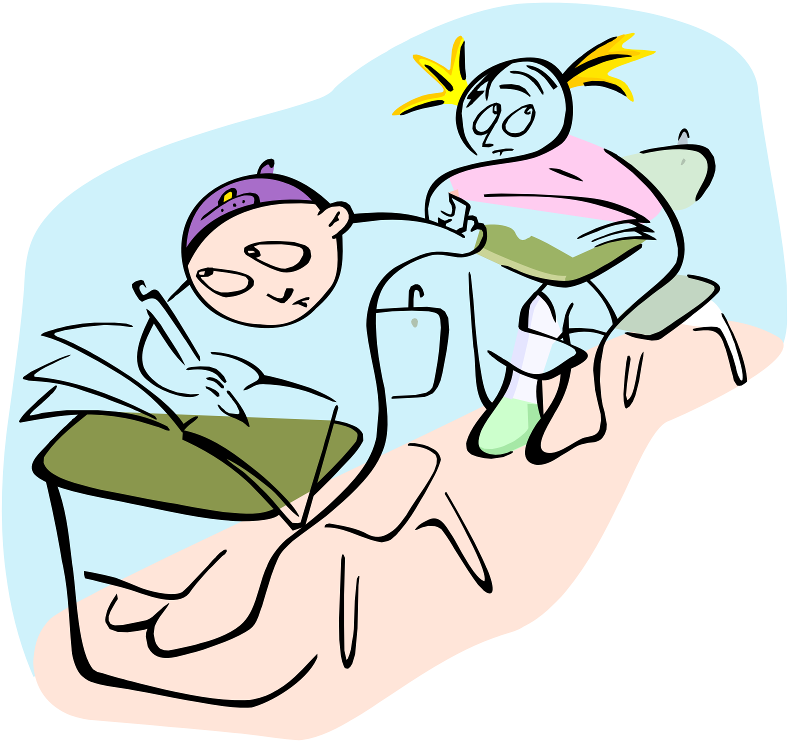

Today’s health education…

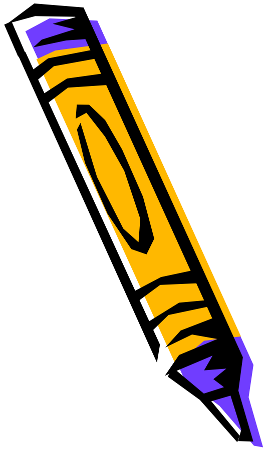

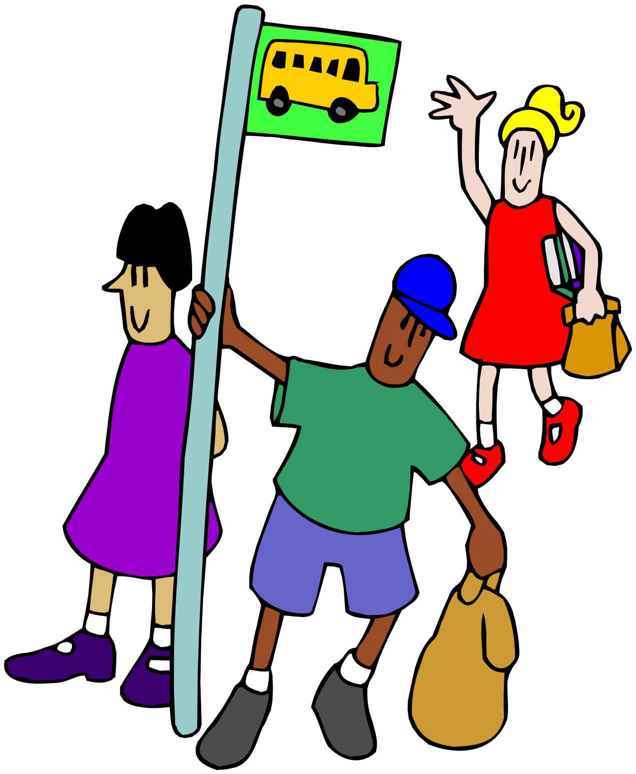

Helps students learn and practice **personal and social skills** to promote and protect health.

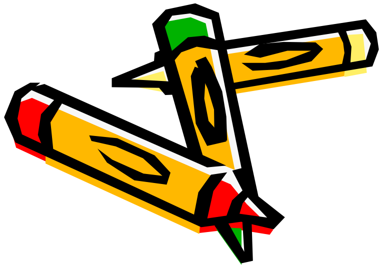

Today's health education…

Who, When, Where?

- *Who*: Students in grades K-12
- *When*: Elementary, middle, and high school
- *Where*: In classrooms, schools, homes, and communities

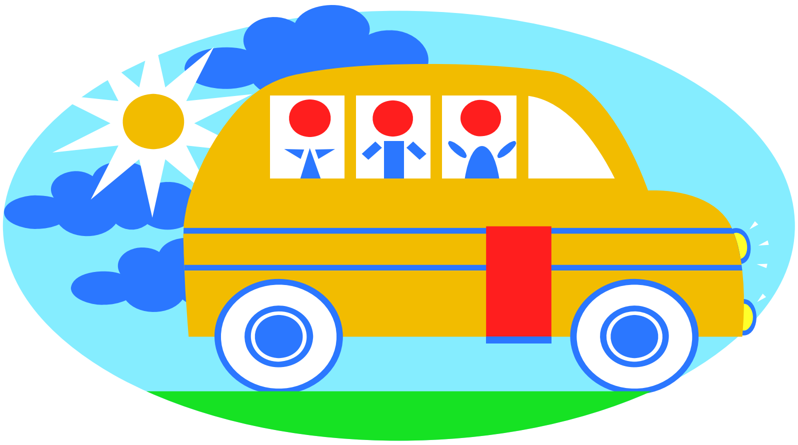

Today's health education…

Why?

Leading Causes of Morbidity and Mortality

Youth (ages 10-24)

1. Motor vehicle crashes
2. Homicide
3. Suicide

Adults (ages 25+)

1. Heart disease
2. Cancer
3. Stroke

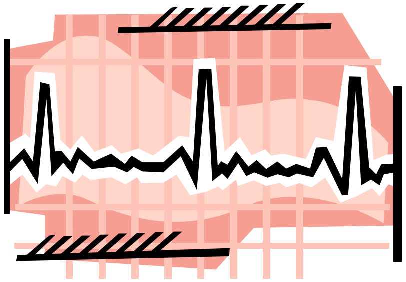

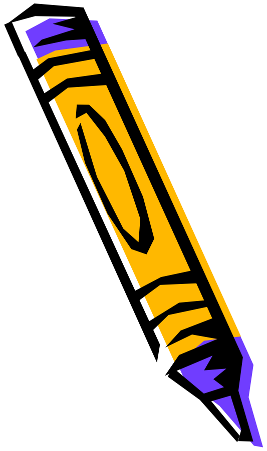

Today’s health education…

Why?

Related Health Risks for Youth

- Alcohol and other drug use
- 800,000 unintended teen pregnancies per year
- 3 million new cases of STDs* among youth each year

*Sexually transmitted diseases.

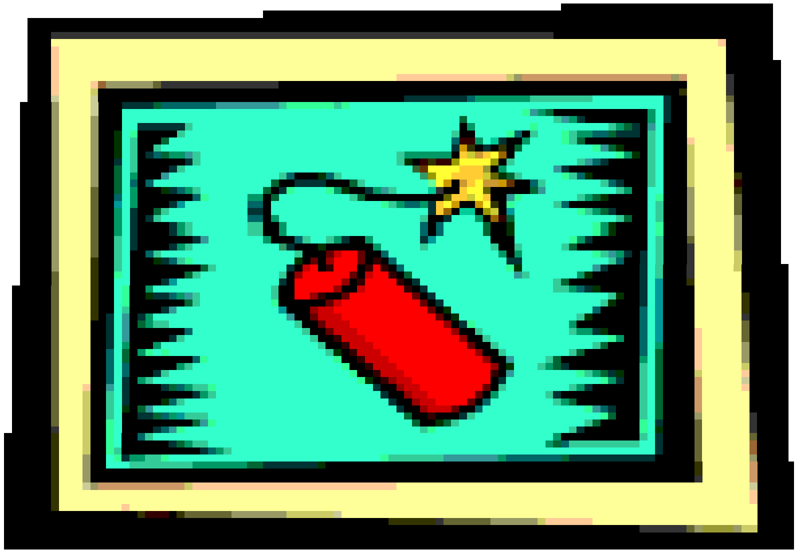

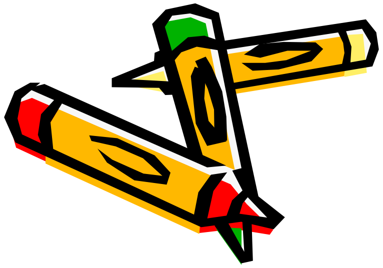

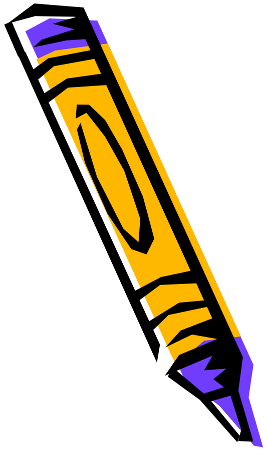

Today's health education…

What?

Education About 7 Priority Content Areas

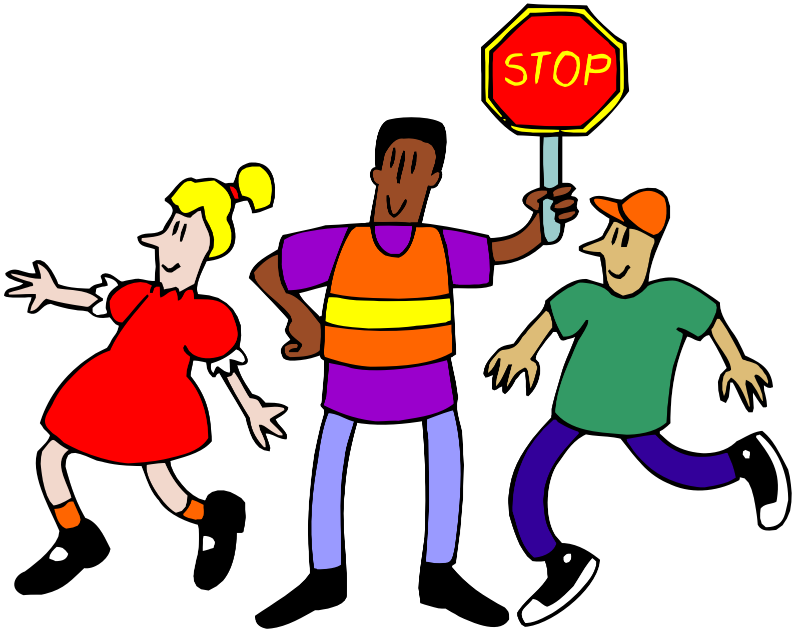

1. Promoting mental and emotional health
2. Promoting healthy eating and physical activity
3. Promoting personal health and wellness
4. Promoting safety and preventing violence
5. Promoting a tobacco-free lifestyle
6. Promoting a lifestyle free of alcohol and other drugs
7. Promoting sexual health and responsibility

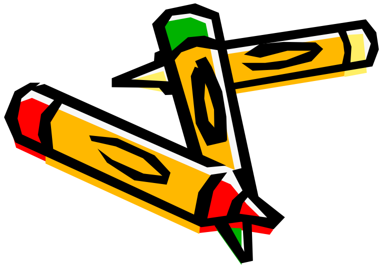

Today's health education…

How?

Developing Personal and Social Skills

1. Core Concepts
2. Accessing Information
3. Self-Management
4. Analyzing Influences
5. Communication
6. Decision Making and Goal Setting
7. Advocacy

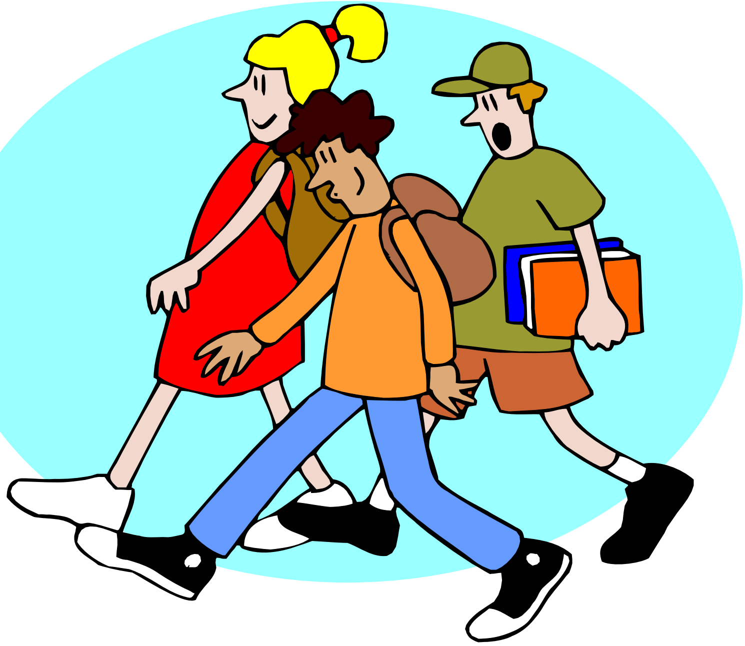

Today’s health education…

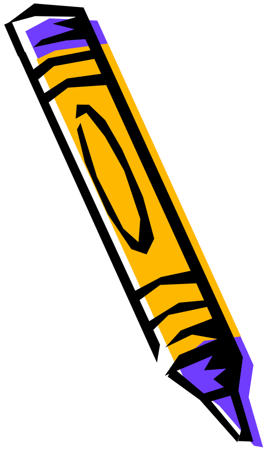

We teach about:

- 7 Personal and Social Skills

AND

- 7 Priority Content Areas

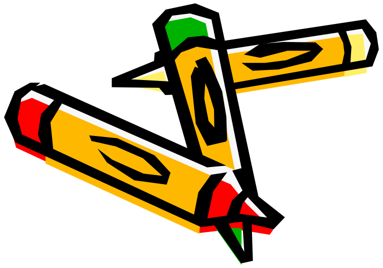

We call this…

Hawaii's 7 by 7 for Health Education

1. Core Concepts
2. Accessing Information
3. Self-Management
4. Analyzing Influences
5. Communication
6. Decision Making and Goal Setting
7. Advocacy

1. Promoting mental and emotional health
2. Promoting healthy eating and physical activity
3. Promoting personal health and wellness
4. Promoting safety and preventing violence
5. Promoting a tobacco-free lifestyle
6. Promoting a lifestyle free of alcohol and other drugs
7. Promoting sexual health and responsibility

Close-Up on Standards:
1. Core Concepts

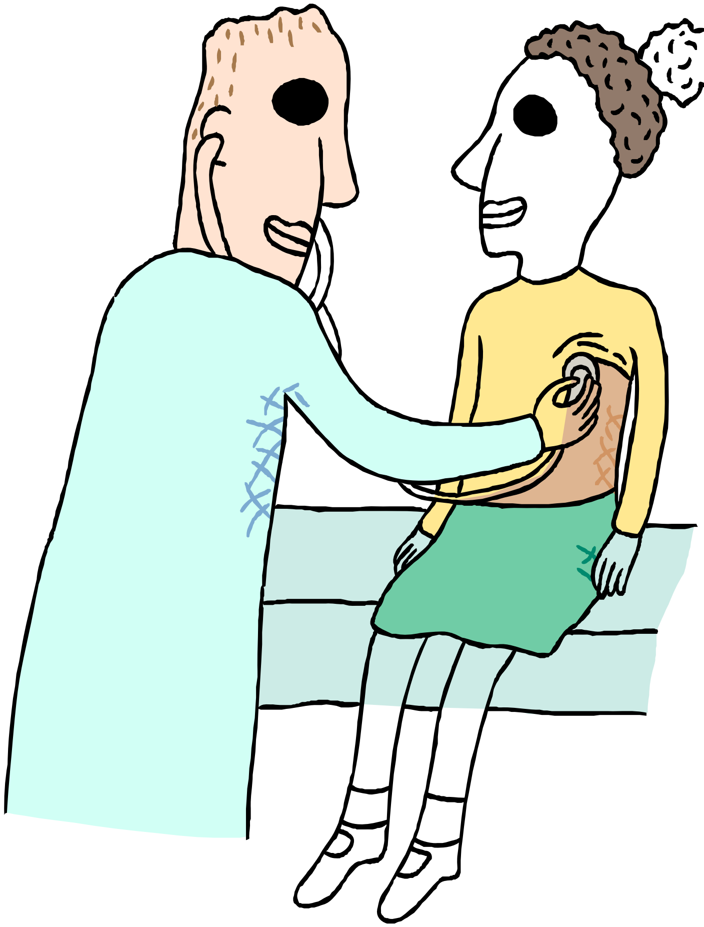

Students will comprehend concepts related to health promotion and disease prevention.

1. Core Concepts: Examples

1. Name the signs of depression.
2. Describe healthy NPA* practices for families.
3. Explain why we don’t touch others’ blood.
4. Explain pedestrian and bicycle safety.
5. List short-term risks of tobacco use.
6. Identify the signs of alcohol poisoning.
7. Describe changes that happen in puberty.

*Nutrition and physical activity.

1. Promoting mental and emotional health
2. Promoting healthy eating and physical activity
3. Promoting personal health and wellness
4. Promoting safety and preventing violence
5. Promoting a tobacco-free lifestyle
6. Promoting a lifestyle free of alcohol and other drugs
7. Promoting sexual health and responsibility

How do we assess Core Concepts?

- Give accurate information.
- Show breadth and depth of knowledge.
- Describe relationships between behavior and health.
- Draw conclusions about connections between behavior and health.

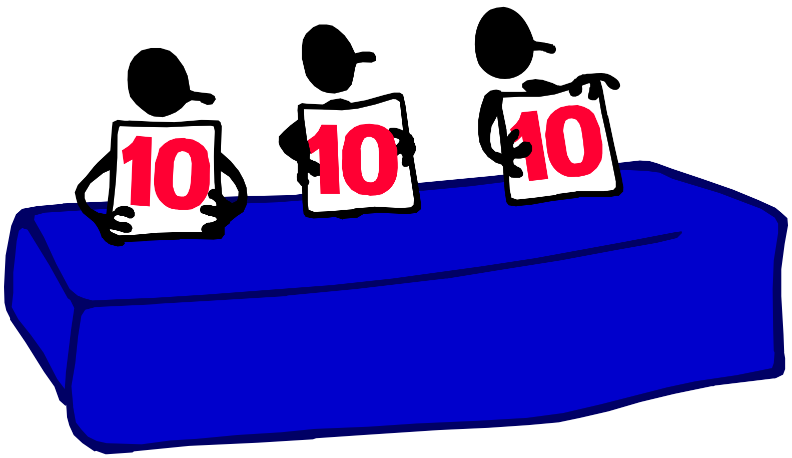

Close-Up on Standards:
2. Accessing Information

Students will access valid health information and health-promoting products and services.

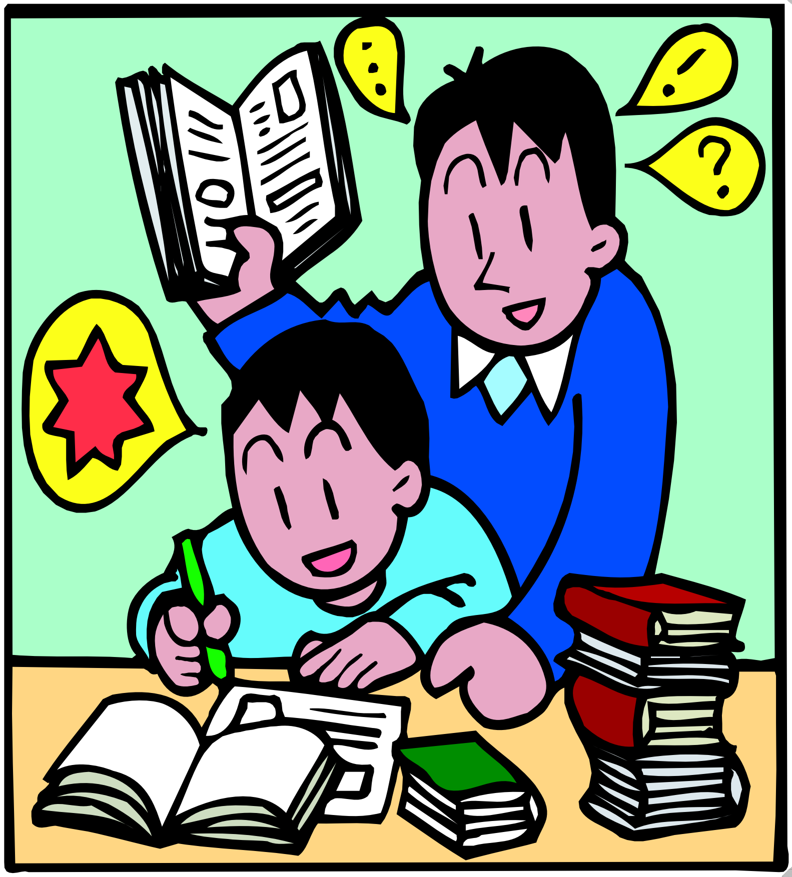

2. Accessing Information: Examples

1. Identify counseling services in community.
2. Compare food labels for favorite snacks.
3. Investigate truthfulness of health claims.
4. Show how to call 911 in emergencies.
5. Find out what's actually in tobacco.
6. List phone numbers for Poison Control Center.
7. Conduct a survey on peers' STD* knowledge.

*Sexually transmitted disease.

1. Promoting mental and emotional health
2. Promoting healthy eating and physical activity
3. Promoting personal health and wellness
4. Promoting safety and preventing violence
5. Promoting a tobacco-free lifestyle
6. Promoting a lifestyle free of alcohol and other drugs
7. Promoting sexual health and responsibility

How do we assess Accessing Information?

- Give the sources for information, products, or services.
- Explain why sources are valid and appropriate.

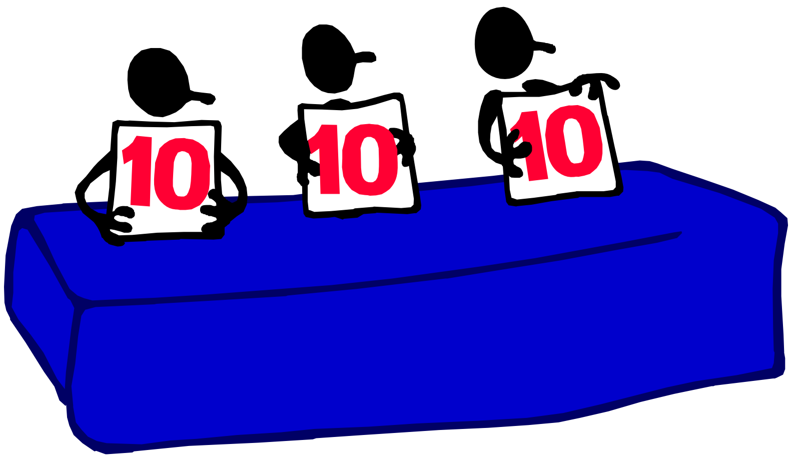

Close-Up on Standards:
3. Self-Management

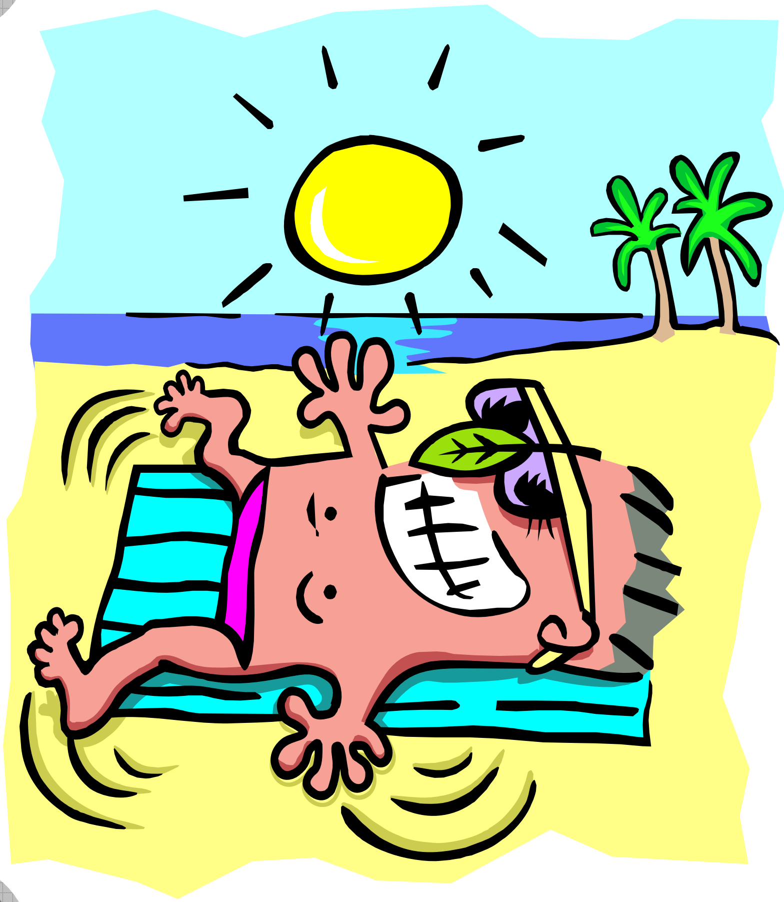

Students will demonstrate the skills to practice healthy behaviors and reduce health risks.

(Stay out of the sun in the middle of the day!)

3. Self-Management: Examples

1. Practice positive self-talk for a whole day.
2. Put athletic gear by the door as a cue for activity.
3. Don’t touch your nose or eyes during flu season.
4. Practice ways to calm down when angry.
5. Do things with other kids who don’t smoke.
6. Stick with friends who don’t use alcohol and other drugs.
7. Avoid situations that may involve pressure for sex.

1. Promoting mental and emotional health
2. Promoting healthy eating and physical activity
3. Promoting personal health and wellness
4. Promoting safety and preventing violence
5. Promoting a tobacco-free lifestyle
6. Promoting a lifestyle free of alcohol and other drugs
7. Promoting sexual health and responsibility

How do we assess Self-Management?

Use a checklist of steps for performing a skill

(Call 911, give information, and stay on the line)

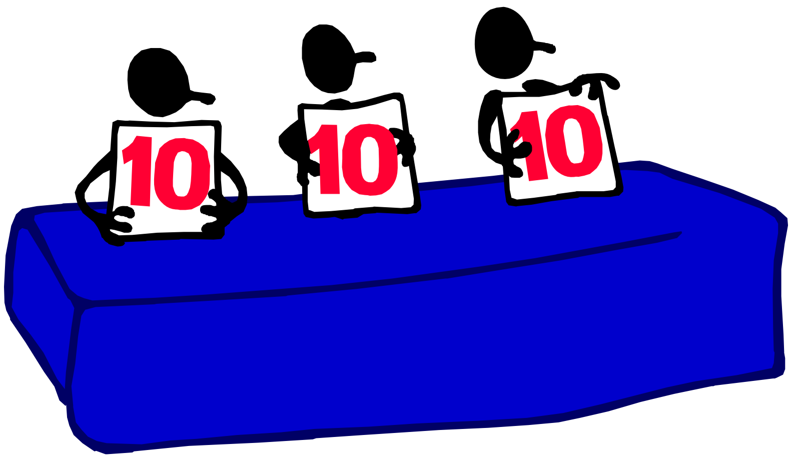

Close-Up on Standards:
4. Analyze Influences

Students will analyze the effects of internal and external influences on health.

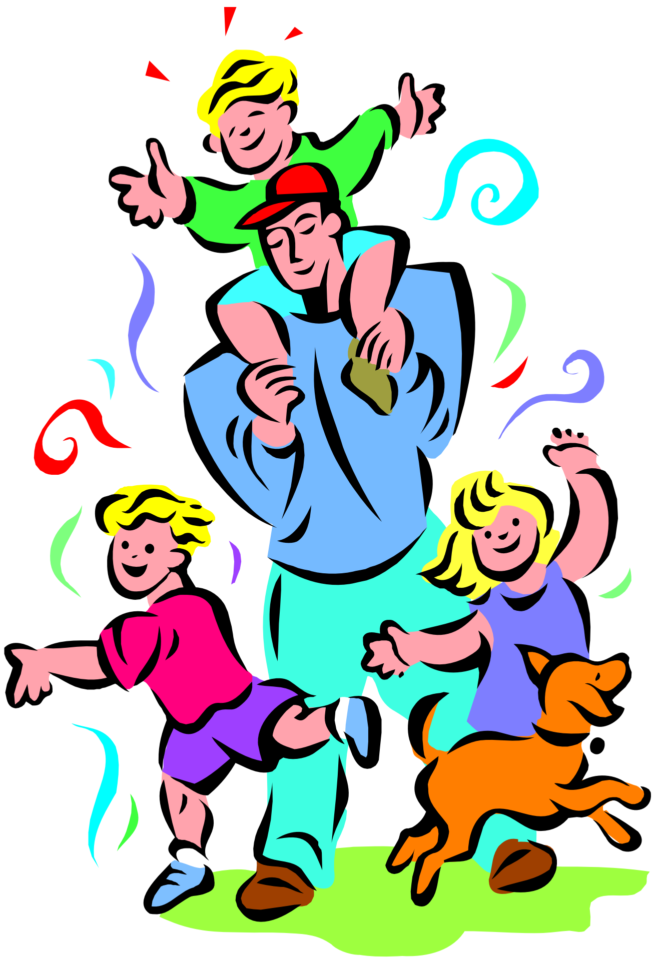

4. Analyze Influences: Examples

1. Find resiliency support in families and culture.
2. Describe favorite family traditions around food.
3. Spot product placement in movies.
4. Describe how violence is portrayed on TV.
5. Analyze tobacco ads, and create “Truth” ads.
6. Demonstrate how peers help prevent use of alcohol and other drugs.
7. Challenge body image messages in magazines.

1. Promoting mental and emotional health
2. Promoting healthy eating and physical activity
3. Promoting personal health and wellness
4. Promoting safety and preventing violence
5. Promoting a tobacco-free lifestyle
6. Promoting a lifestyle free of alcohol and other drugs
7. Promoting sexual health and responsibility

How do we assess Analyze Influences?

- Describe internal and external influences on health
- Explain how influences can affect health decisions
- Tell how influences can work for and against each other

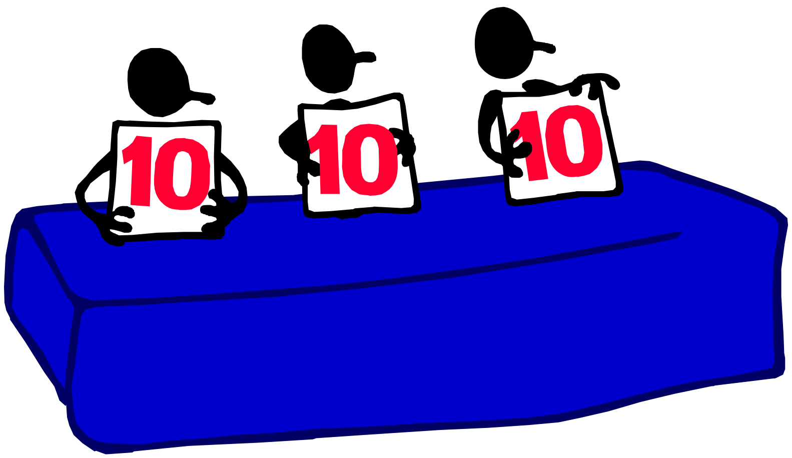

Close-Up on Standards:
5. Interpersonal Communication

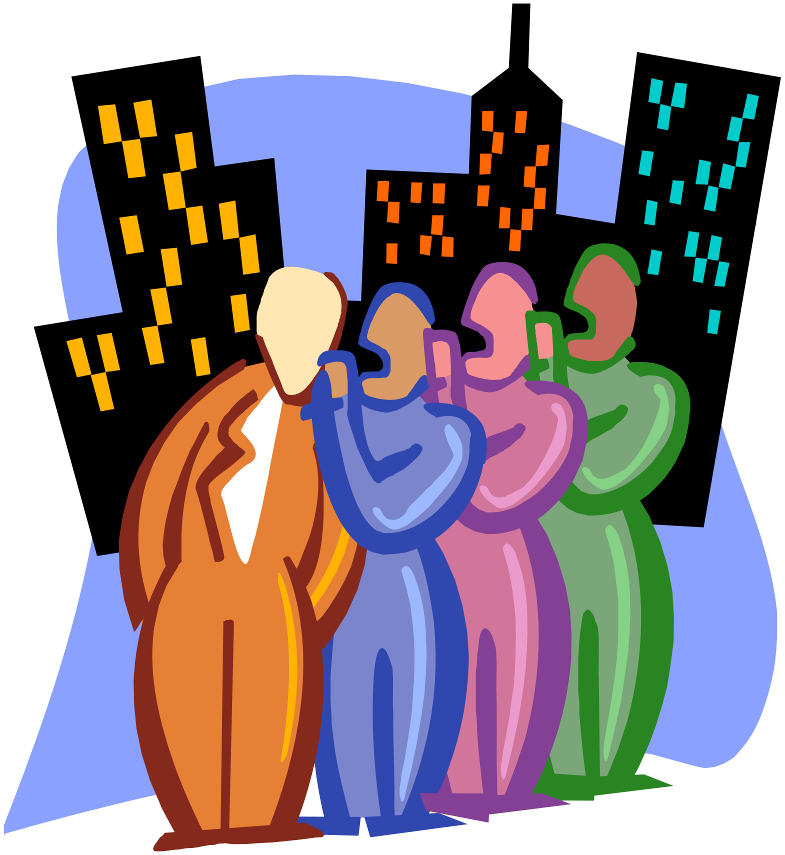

Students will use interpersonal communication skills to enhance health.

5. Interpersonal Communication: Examples

1. Promoting mental and emotional health
2. Promoting healthy eating and physical activity
3. Promoting personal health and wellness
4. Promoting safety and preventing violence
5. Promoting a tobacco-free lifestyle
6. Promoting a lifestyle free of alcohol and other drugs
7. Promoting sexual health and responsibility

1. Listen to a friend who is upset or discouraged.
2. Gently let Grandma know you are full already!
3. Tell a trusted adult about uncomfortable situations.
4. Talk with an angry friend to help calm a situation.
5. Use humor to say no to tobacco use.
6. Suggest alternatives to scenarios involving use of alcohol and other drugs.
7. Talk with adult family members about dating.

How do we assess Interpersonal Communication?

- Use appropriate verbal and nonverbal messages.
- Use skills such as negotiation, refusal, and conflict management.
- Use strategies such as "I" messages, eye contact, tone of voice, body language, and repeated refusals.

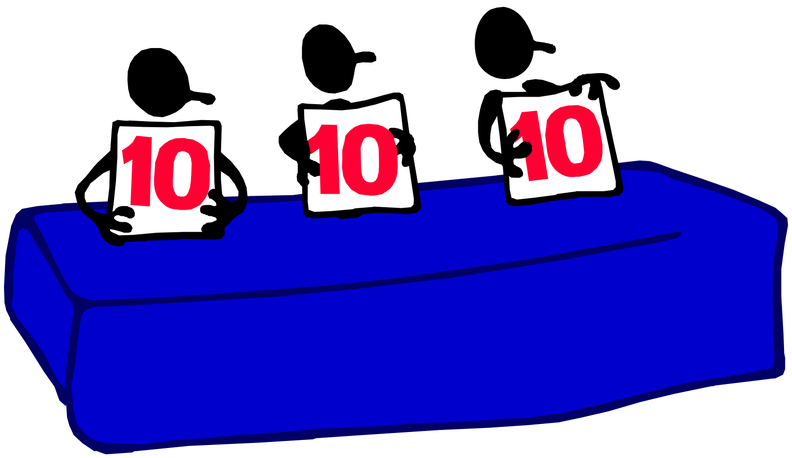

Close-Up on Standards: 6. Decision Making and Goal Setting

Students will use decision-making and goal-setting skills to enhance health.

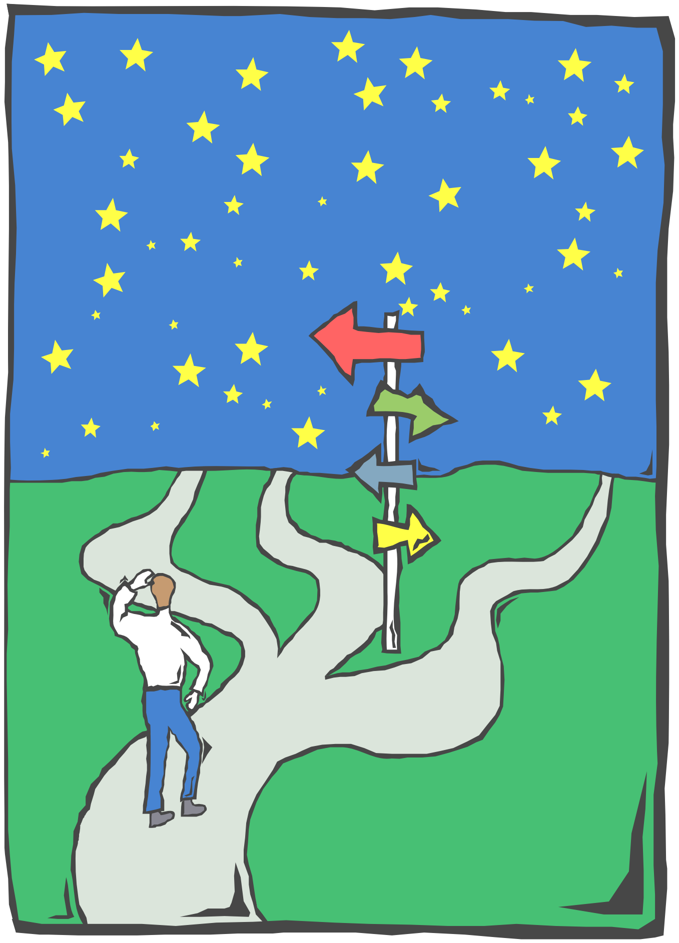

6. Decision Making and Goal Setting: Examples

1. Promoting mental and emotional health
2. Promoting healthy eating and physical activity
3. Promoting personal health and wellness
4. Promoting safety and preventing violence
5. Promoting a tobacco-free lifestyle
6. Promoting a lifestyle free of alcohol and other drugs
7. Promoting sexual health and responsibility

1. Remember: “I think I can, I think I can!”
2. Set nutrition and activity goals with family members.
3. Keep chart to track personal goal progress.
4. Set a weekly class goal of no fighting at school.
5. Support family member who wants to quit smoking.
6. Describe decision steps to avoid alcohol and other drugs at parties.
7. Explain goals that don’t include teen pregnancy.

How do we assess Decision Making and Goal Setting?

- Identify the problem, state alternatives, give consequences, take action, and evaluate the outcome.
- Design a clear goal statement and a plan that includes logical steps, ways to build support and deal with obstacles, and strategies to assess progress.

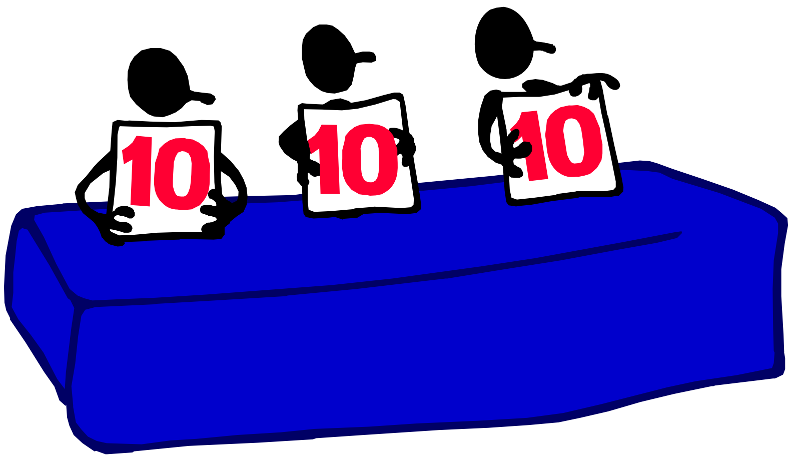

Close-Up on Standards:
7. Advocacy

Students will advocate for personal, family, and community health.

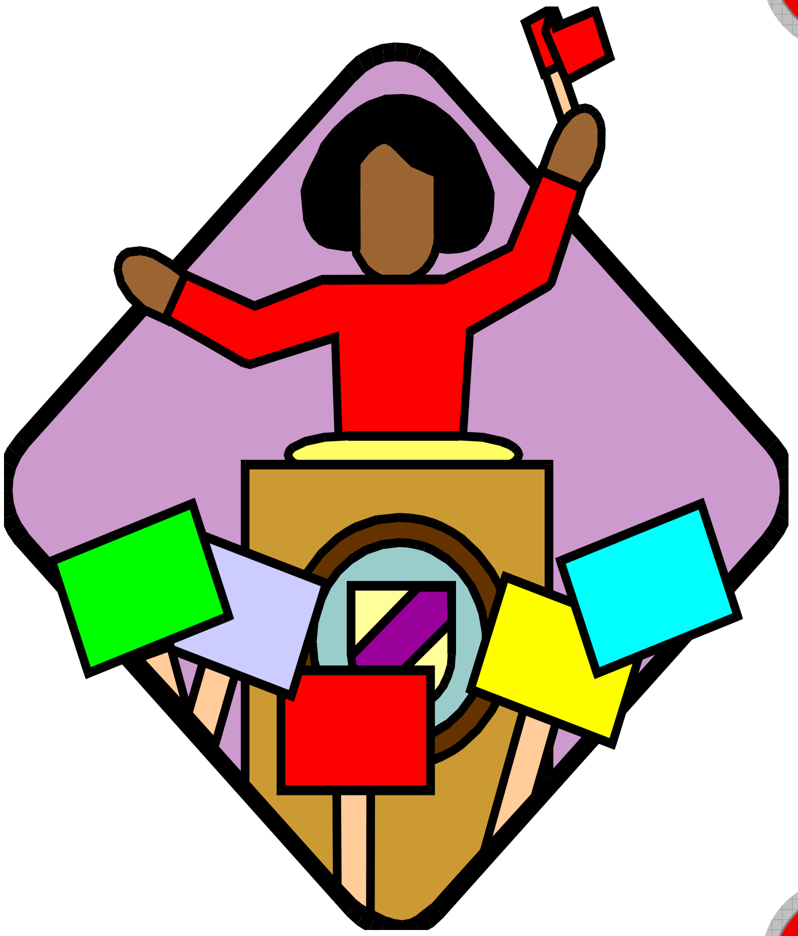

7. Advocacy: Examples

1. Promoting mental and emotional health
2. Promoting healthy eating and physical activity
3. Promoting personal health and wellness
4. Promoting safety and preventing violence
5. Promoting a tobacco-free lifestyle
6. Promoting a lifestyle free of alcohol and other drugs
7. Promoting sexual health and responsibility

1. Publicize ways to manage stress during exam week.
2. Design a healthy eating and activity school newsletter.
3. Arrange for a student and family first aid class at school.
4. Carry out a No Name-Calling Week campaign.
5. Write letters of support to tobacco-free restaurants.
6. Support friends who choose not to use alcohol and other drugs.
7. Design a sexual health and responsibility campaign.

How do we assess Advocacy?

- State a clear, health-enhancing position.
- Back it up with facts and data.
- Target the audience.
- Express strong conviction for position.

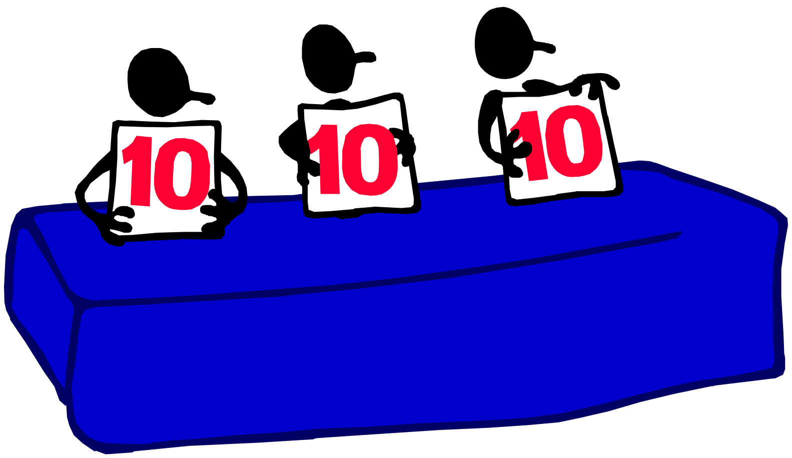

How do I start teaching?

Have students build skills for positive health habits:

- Promote mental and emotional health.
- Promote healthy eating and physical activity.
- Promote personal health and wellness.

Help students adapt their skills to manage health risks:

- Promote safety and prevent violence.
- Promote a tobacco-free lifestyle.
- Promote a lifestyle free of alcohol and other drugs.
- Promote sexual health and responsibility.

What resources are available?

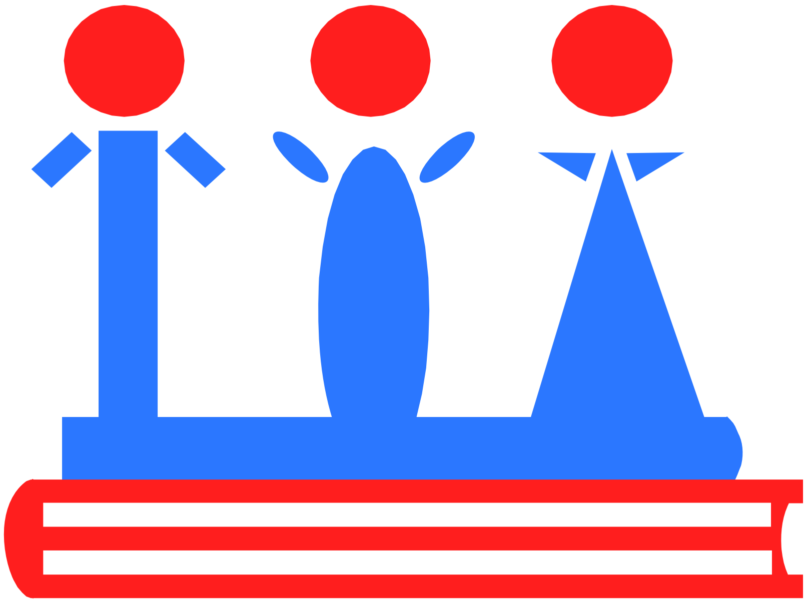

- Professional development from state and local education agencies
- Health Education Curriculum Analysis Tool (HECAT), CDC*
- Data from Youth Risk Behavior Survey (YRBS) and Youth Tobacco Survey (YTS), CDC*
- Rocky Mountain Center for Health Promotion and Education (www.rmc.org)
- Web sites for kids (e.g., BAM! Body and Mind, www.bam.gov)
- Web sites for teachers (e.g., HealthTeacher, www.healthteacher.com)

*Centers for Disease Control and Prevention.

Dive into health education!

Contact your state and local education agencies.

Contact your local health department.

Contact the Division of Adolescent and School Health, Centers for Disease Control and Prevention (CDC) at www.cdc.gov/HealthyYouth/.

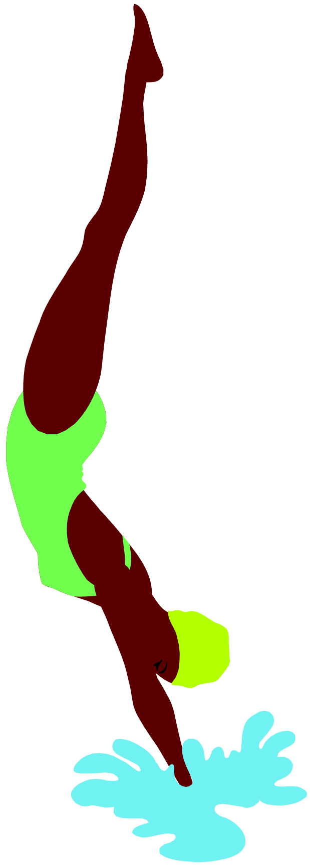

For more information on Hawaii's "7 by 7," contact:

Beth Pateman, HSD, MPH
University of Hawaii at Manoa
(808) 956-3995
mpateman@hawaii.edu
